# Case Report: Pulmonary nocardiosis: three case reports and literature review

**DOI:** 10.3389/fmed.2026.1789986

**Published:** 2026-03-18

**Authors:** Qi Zhao, Min Wang, Xincheng Nie, LIna Fu, Xuehao Fu

**Affiliations:** 1 Shandong University of Traditional Chinese Medicine, Jinan, China; 2 The Second Affiliated Hospital of Shandong University of Traditional Chinese Medicine, Jinan, China

**Keywords:** bronchoscopy, case report, combination therapy, next-generation sequencing, prognosis, pulmonary nocardiosis

## Abstract

Pulmonary nocardiosis is a relatively uncommon opportunistic infection characterized by nonspecific clinical features, often leading to delays in diagnosis and treatment. We describe the clinical data of three confirmed cases of pulmonary *Nocardia* infection. We also systematically reviewed relevant literature from the PubMed database between January 2015 and September 2025, and performed a pooled analysis of 119 patients. Case 1 involved an elderly patient with bronchiectasis, diagnosed via targeted next-generation sequencing (tNGS) of sputum, who showed significant radiological improvement after treatment with trimethoprim-sulfamethoxazole. Case 2, with coexisting bronchiectasis and chronic obstructive pulmonary disease, was diagnosed by tNGS of bronchoalveolar lavage fluid and switched to linezolid-based combination therapy due to drug intolerance. Case 3 involved a patient with underlying structural lung disease who experienced protracted infection but eventually achieved control following individualized combination antimicrobial therapy and supportive care. Literature analysis revealed a median age of 57 years with a male predominance (55.5%). Common comorbidities included chronic lung diseases (39.5%) and immunosuppression-related conditions (39.5%). Chest CT findings mainly featured consolidation (47.9%) and nodules (41.2%). Diagnosis relied primarily on sputum/airway secretions (48.7%) and bronchoalveolar lavage fluid (42.9%), and 83.2% of patients received regimens containing trimethoprim-sulfamethoxazole. Patients undergoing next-generation sequencing and/or bronchoscopy showed a trend toward lower mortality. This report suggests that a precision pathogen diagnosis strategy centered on bronchoscopy and next-generation sequencing, combined with susceptibility-guided and dynamically adjusted individualized combination antimicrobial therapy, may help improve clinical outcomes in patients with pulmonary nocardiosis. Close monitoring and management of adverse drug reactions are essential during long-term treatment.

## 1 Introduction

Pulmonary nocardiosis is a relatively rare clinical infection caused by aerobic actinomycetes of the genus *Nocardia*, typically presenting with non-specific symptoms. Conventional pathogen detection methods often lack sensitivity, frequently leading to diagnostic and therapeutic delays, which can result in increased mortality and pose a significant challenge in clinical practice. *Nocardia* species are ubiquitous environmental saprophytes found in soil, water, decaying vegetation, and organic matter (1). As classic opportunistic pathogens, they primarily infect individuals who are immunocompromised. The main route of infection is respiratory inhalation, with the lungs being the most common site of disease (2). Treatment is prolonged, available antimicrobial options are limited, and adverse reactions are common. In recent years, the reported incidence of nocardiosis has shown an upward trend, likely due to the widespread use of immunosuppressive agents and advancements in diagnostic techniques (3).

We report three confirmed cases of pulmonary nocardiosis and conduct a systematic review of the literature from the PubMed database between January 2015 and September 2025. The clinical features, imaging findings, and diagnostic and therapeutic strategies are analyzed.

## 2 Case presentation

### 2.1 Case 1

**Figure 1:**
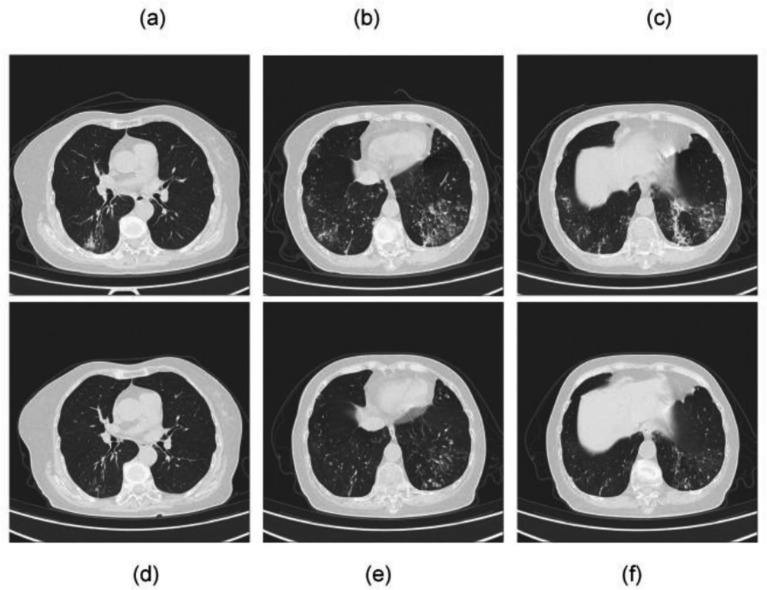
Chest CT scans of Case 1. Prior to treatment initiation: **(a–c)** multiple ill-defined focal consolidations are observed, accompanied by diffusely increased lung markings and ground-glass opacities in both lungs. One month after treatment: **(d–f)** the previously noted consolidations have significantly decreased in size and density, with partial resolution of ground-glass opacities.

A 68-year-old female was admitted on April 28, 2025, with a chief complaint of cough and expectoration for over 10 years. These symptoms had exacerbated for 3 days prior to admission, accompanied by hemoptysis and fever for 1 day. The patient experienced an exacerbation of cough and expectoration without apparent trigger 3 days prior to admission, accompanied by clear rhinorrhea. She self-medicated with “Ganmaoqing Granules and Cefradine” without symptomatic improvement. She developed a fever yesterday, with a peak temperature of 38.6 °C, accompanied by chills, headache, and myalgia, but without rigors. A single episode of hemoptysis was noted this morning. The patient has a 10-year history of bronchiectasis, a 7-year history of hypertension, well-controlled on oral amlodipine. In 2023, the patient underwent bronchoscopy at another hospital, where bronchoalveolar lavage fluid culture grew Aspergillus flavus. Consequently, the patient received antifungal therapy with voriconazole and subsequently isavuconazole for 3 months, which was eventually discontinued due to liver function abnormalities. The patient had no history of diabetes mellitus or tuberculosis, and no history of long-term glucocorticoid or immunosuppressant use. The patient has a history of exposure to soil and flower fertilizer. Physical examination revealed bilateral coarse breath sounds and scattered moist rales. Vital signs on admission were stable: body temperature 36.2 °C; pulse rate 80 beats per minute; respiratory rate 22 breaths per minute; blood pressure 139/72 mmHg. Initial laboratory studies indicated an active infectious and inflammatory process: total white blood cell count: 12.43 × 10^9^/L (reference range: 3.5–9.5 × 10^9^/L), Neutrophil percentage: 81.4% (reference range: 40–75%), C-reactive protein (CRP): 118.7 mg/L (reference range: 0–1.6 mg/L). Hypokalemia (2.60 mmol/L) and hypoalbuminemia (37.5 g/L) were also present. Chest CT findings were consistent with infected bronchiectasis. Multiple ill-defined focal consolidations were observed, accompanied by diffusely increased lung markings and ground-glass opacities in both lungs (Figure 1). Pulmonary function tests indicated moderate to severe mixed ventilatory dysfunction.

**Figure 2:**
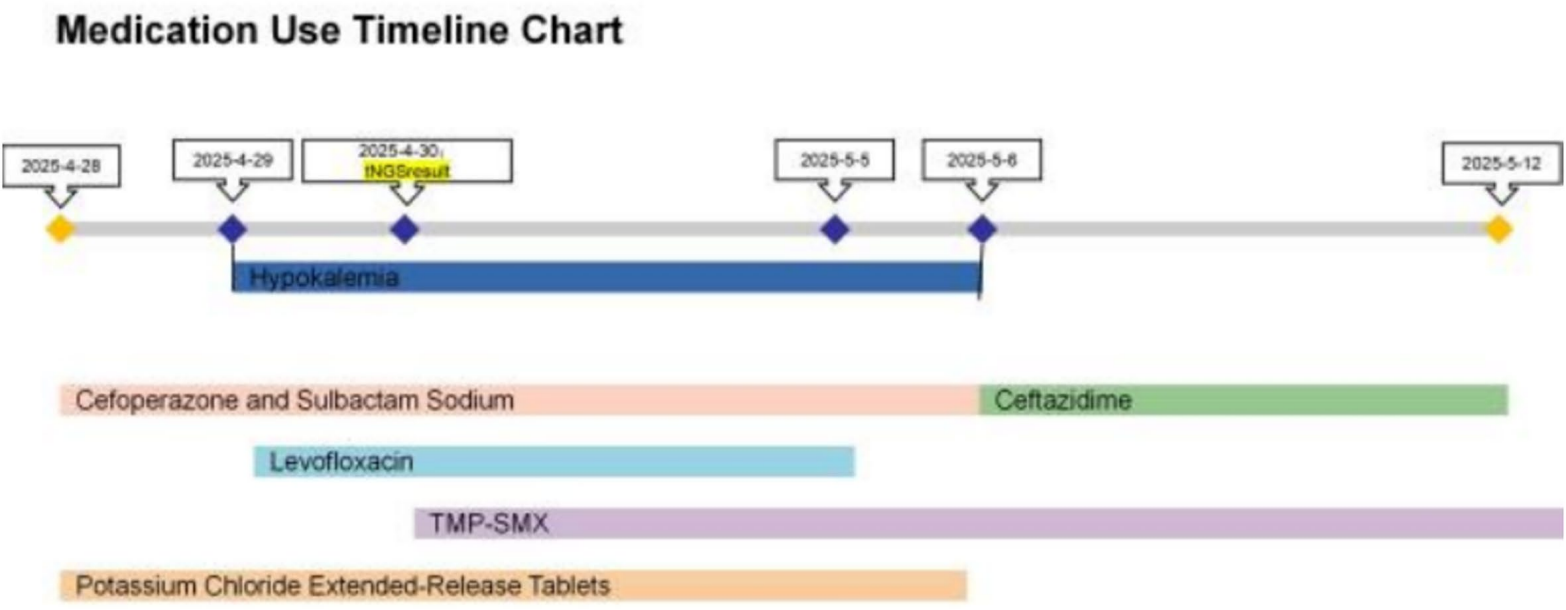
Medication chart for Case 1.

Empirical therapy with cefoperazone-sulbactam and levofloxacin was initiated. The patient has a history of bronchiectasis complicated by previous Aspergillus infection. She/he presented with hemoptysis upon admission and had a history of exposure to soil and flower fertilizer, leading to a high clinical suspicion of infection with such pathogens. Therefore, both routine sputum culture and tNGS were performed on the day of admission to rapidly identify the causative agent. On April 29, the tNGS results revealed a co-infection with *Nocardia* spp. (sequence count: 689) and *Moraxella catarrhalis* (sequence count: 83,936). On May 2, routine culture and antimicrobial susceptibility testing results were reported. Sputum smear Gram stain revealed moderate Gram-negative cocci with evidence of phagocytosis by leukocytes. Haemophilus culture showed no pathogenic growth, and fungal culture was negative. Bacterial culture confirmed the presence of *Moraxella catarrhalis*. Antimicrobial susceptibility testing demonstrated that the isolated *M. catarrhalis* strain was susceptible to trimethoprim-sulfamethoxazole, cefotaxime, ceftazidime, and multiple other antimicrobial agents, and exhibited intermediate susceptibility to clindamycin. Based on the susceptibility profile, therapy was adjusted on May 2, 2025. Treatment for *Nocardia* was initiated with trimethoprim-sulfamethoxazole (TMP-SMX) at a dose of 3 tablets three times daily, while antibiotics were switched to ceftazidime. Due to gastrointestinal intolerance, the TMP-SMX dose was reduced to two tablets TID, and levofloxacin was discontinued due to intolerance. Following these adjustments, her cough and sputum production improved significantly, and she was discharged on May 12, 2025. On June 13, 2025, follow-up chest CT demonstrated significant reduction in size and density of the previously noted consolidations, along with partial resolution of ground-glass opacities, indicating a positive response to therapy (Figure 1). The treatment timeline is illustrated in Figure 2.

### 2.2 Case 2

**Figure 3:**
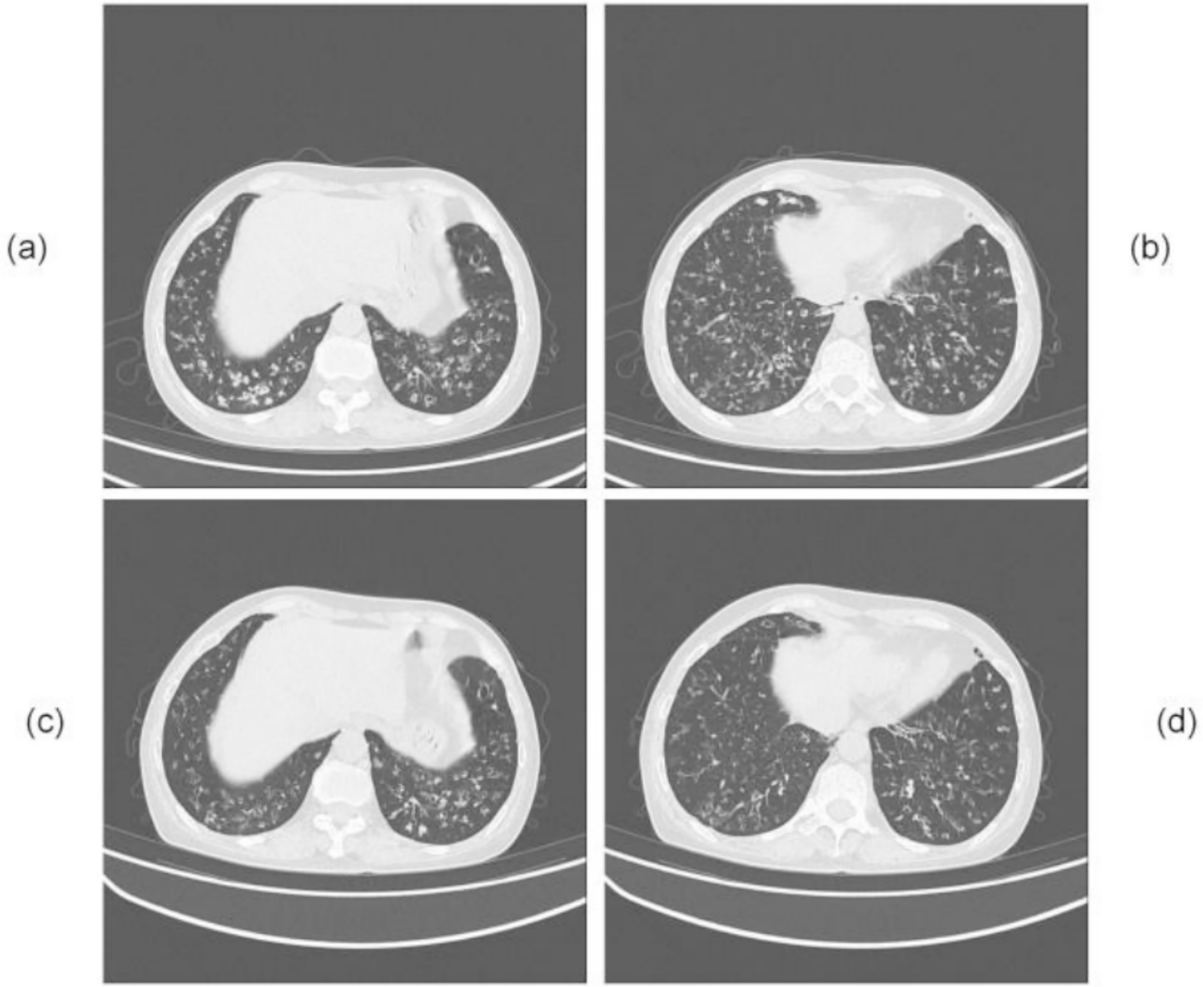
Chest CT of Case 2. Prior to treatment initiation: **(a,b)** multiple nodular opacities are seen in both lung fields, more prominent in the right lung, accompanied by distorted lung markings and extensive, heterogeneous patchy consolidations and ground-glass opacities in the subpleural regions. One month after treatment. **(c,d)** The number and extent of bilateral nodular opacities have significantly decreased. Marked improvement is noted in the distorted lung markings of the right subpleural area, demonstrating overall definite improvement compared to the pretreatment scan.

A 54-year-old female was admitted on June 5, 2025, due to “cough with yellow-green sputum for over 8 years, and hemoptysis for 3 days.” The patient developed hemoptysis 3 days prior to admission, with dark red blood of approximately 50 mL, accompanied by fatigue and pharyngalgia. Oral administration of “levofloxacin, Yunnan Baiyao, and traditional Chinese medicine decoction” yielded minimal effect. Before the onset of illness, the patient had been exposed to flower fertilizer. She has a long-standing history of bronchiectasis and chronic obstructive pulmonary disease, with recurrent cough and yellow-green sputum in recent years, and episodes of hemoptysis occurring 4–5 times annually, each involving a small volume of blood. She has been on long-term oral traditional Chinese medicine treatment and reports a history of allergy to penicillins and cephalosporins. She denies any history of hypertension, diabetes mellitus, tuberculosis, or other chronic conditions, and has no history of long-term glucocorticoid or immunosuppressant use. Physical examination revealed diminished breath sounds and scattered moist rales bilaterally. Vital signs on admission were stable: Temperature 36.4 °C; pulse rate 92 beats per minute; respiratory rate 19 breaths per minute; blood pressure 127/75 mmHg. Initial laboratory tests did not show significant leukocytosis but indicated inflammation: ESR: 32 mm/h↑ (reference range: 0–15 mm/h), CRP: 7.6 mg/L↑ (reference range: 0–1.6 mg/L), Total white blood cell count: 5.24 × 10^9/L (reference range: 3.5–9.5 × 10^9/L), Neutrophil percentage: 57.9% (reference range: 40–75%). Chest CT revealed inflammatory changes with bronchiectasis in both lungs. Multiple nodular opacities were observed in both lung fields, more prominent in the right lung, accompanied by distorted lung markings and extensive, heterogeneous patchy consolidations and ground-glass opacities in the subpleural regions (Figure 3). Pulmonary function tests demonstrated severe mixed ventilatory dysfunction.

The initial empirical treatment was intravenous meropenem combined with amikacin. On June 6, sputum specimens tested negative for pathogens via acid-fast staining, bacterial culture, and fungal culture. The patient’s imaging findings revealed multiple nodules with a subpleural distribution in both lungs, a pattern highly suggestive of atypical pathogen infection. The negative results of routine microbiological tests stood in stark contrast to the pronounced imaging abnormalities. Given this discrepancy, along with the presence of hemoptysis, a high clinical suspicion was raised for an atypical pathogen infection. Fiberoptic bronchoscopy with saline lavage of the left lower lobe was performed on June 9, 2025. Routine microbiological examinations of the bronchoalveolar lavage fluid specimen, including acid-fast staining, bacterial culture, and fungal culture, all tested negative for pathogens. However, analysis of the bronchoalveolar lavage fluid by tNGS identified *Pseudomonas aeruginosa* (17,182 sequences; 86.4% relative abundance) and *Nocardia* spp. (2,703 sequences; 12.3%). On June 10, a follow-up sputum specimen again tested negative for pathogens via acid-fast staining, bacterial culture, and fungal culture. Accordingly, TMP-SMX was added on June 13, 2025, to target the *Nocardia* infection. However, the patient experienced significant adverse reactions, including nausea, dizziness, and generalized weakness, leading to discontinuation of TMP-SMX the following day. The regimen was subsequently switched to intravenous linezolid combined with levofloxacin, while intravenous amikacin was discontinued and replaced with nebulized inhalation for adjunctive therapy. This change resulted in the resolution of hemoptysis, symptomatic improvement, and stabilized markers, allowing for discharge. On July 31, 2025, follow-up chest CT demonstrated a significant reduction in the number and extent of bilateral nodular opacities. Significant resolution of the consolidations and ground-glass opacities in the right subpleural regions was observed, along with notable improvement of the distorted pulmonary markings, demonstrating a definite overall improvement compared to the pretreatment scan (Figure 3).

**Figure 4:**
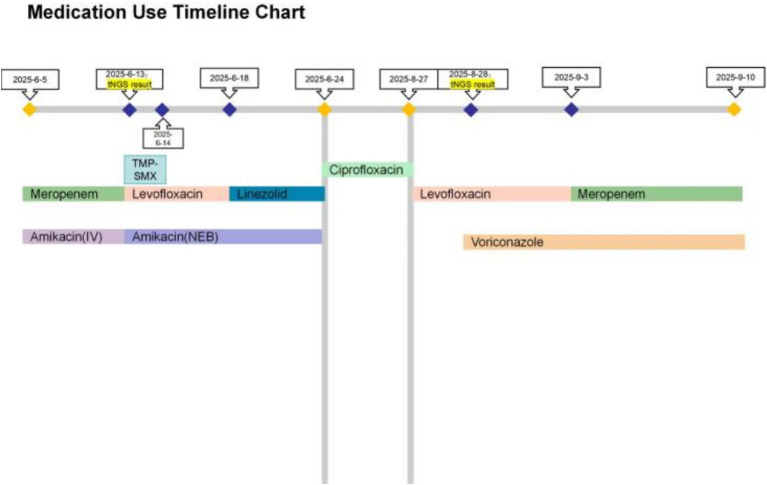
Medication chart for Case 2.

The patient was readmitted on August 27, 2025, with fever and yellow-green sputum. Inflammatory markers were markedly elevated: ESR: 84 mm/h (reference range: 0–15 mm/h), C-reactive protein 77.0 mg/L (reference range: 0–1.6 mg/L), Total white blood cell count: 4.99 × 10^9/L (reference range: 3.5–9.5 × 10^9/L), Neutrophil percentage: 57.9% (reference range: 40–75%). A chest CT scan confirmed persistent bilateral inflammatory lesions and bronchiectasis. On August 28, 2025, a follow-up sputum specimen was analyzed by tNGS, which detected *Aspergillus niger* (sequence count: 48,916) and *Aspergillus fumigatus* (sequence count: 1,547), with no *Nocardia* detected. Subsequently, on September 2, 2025, sputum culture grew *Aspergillus flavus*, leading to a supplementary diagnosis of pulmonary aspergillosis. At this juncture, the patient had completed approximately 2 months of anti-*Nocardia* therapy, with significant clinical and radiological improvement, and *Nocardia* was no longer detected by NGS, suggesting that the *Nocardia* infection had been effectively controlled. Given that aspergillosis had become the predominant issue, the anti-infective regimen was adjusted to meropenem combined with voriconazole, which led to significant symptomatic improvement, and a decrease in inflammatory markers (CRP 6.6 mg/L). The patient was discharged on September 10, 2025. The medication timeline for Case 2 is summarized in Figure 4.

### 2.3 Case 3

A 65-year-old female was admitted on June 25, 2024, due to “recurrent cough and expectoration for over 9 years, exacerbated for 7 days.” The patient experienced an exacerbation of cough and expectoration 7 days prior to admission, with a 1-day episode of blood-tinged sputum. She developed a fever 5 days ago, with a peak temperature of 37.8 °C. Oral administration of levofloxacin tablets and Suhuang Zhike Capsules resulted in resolution of the fever, but the cough persisted. A chest CT performed at another hospital revealed bilateral bronchiectasis with new-onset inflammation in the left lung. The patient was subsequently referred to our hospital for further management. She has a 9-year history of bronchiectasis, characterized by recurrent cough, expectoration, and blood-tinged sputum, with multiple hospitalizations at other institutions for anti-infective therapy. Four years ago, due to a positive T-cell test for infection, she underwent sputum and bronchoalveolar lavage fluid examinations for acid-fast bacilli at another hospital, both of which were negative. Subsequently, she received empirical anti-tuberculosis therapy for 2 years, specific medications unknown. In 1996, she underwent bowel resection for ulcerative colitis, with recurrent postoperative intestinal obstructions. In 2001, she underwent a right thyroidectomy and has since maintained normal thyroid function. The patient reported no history of allergy to penicillins and cephalosporins prior to surgery but developed an allergy postoperatively. She denies any history of hypertension, diabetes mellitus, or other chronic conditions, and has no history of long-term glucocorticoid or immunosuppressant use. The patient reported a history of soil contact. Vital signs on admission were stable. Examination revealed wet rales in the right lower lung field. Vital signs on admission were: temperature 36.5 °C, pulse rate 64 beats per minute, respiratory rate 18 breaths per minute, and blood pressure 129/71 mmHg. Laboratory tests indicated significant inflammation: CRP: 10.1 mg/L (reference range:0–1.6 mg/L), Erythrocyte sedimentation rate: 38 mm/h (reference range: 0–15 mm/h), Total white blood cell count: 6.07 × 10^9/L (reference range: 3.5–9.5 × 10^9/L), Neutrophil percentage: 59.1% (reference range: 40–75%). Upon admission, sputum bacterial and fungal cultures both tested negative for pathogens.

The patient presented with hemoptysis and fever. Routine sputum cultures were negative, and her symptoms did not fully resolve following empirical levofloxacin therapy. Given her long-standing history of bronchiectasis and the protracted disease course, a high clinical suspicion for atypical pathogen infection was raised. Consequently, bronchoscopy was performed on June 28, and bronchoalveolar lavage fluid (BALF) was sent for microbiological culture, antimicrobial susceptibility testing (AST), and targeted next-generation sequencing (tNGS). BALF smear revealed the presence of weakly acid-fast bacilli, while fungal culture was negative. Bacterial culture of the BALF grew *Pseudomonas aeruginosa* and *Nocardia abscessus*. AST results showed that the isolated *P. aeruginosa* strain was susceptible to levofloxacin, ceftazidime, and carbapenems, and demonstrated intermediate susceptibility to amikacin. The *N. abscessus* strain was susceptible to trimethoprim-sulfamethoxazole, amikacin, and linezolid, but resistant to ciprofloxacin. On July 2, tNGS results further confirmed a co-infection with *Nocardia abscessus* and *Pseudomonas aeruginosa*. Initial therapy, guided by antimicrobial susceptibility, consisted of intravenous amikacin and levofloxacin, followed by sequential oral TMP-SMX. Following symptomatic improvement, discharge took place on July 8, 2024.

**Figure 5:**
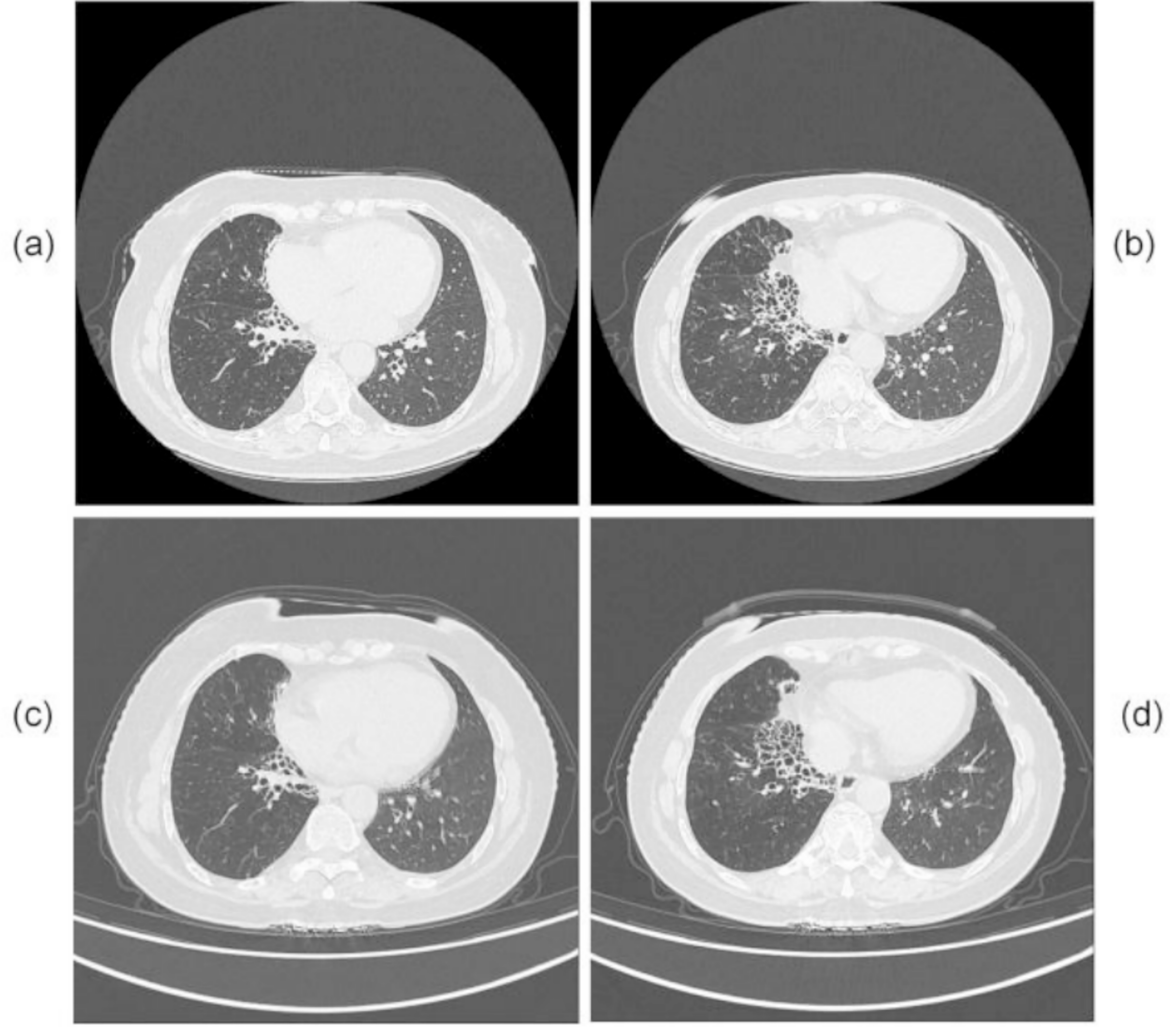
Chest CT of Case 3. After 2 months of treatment: **(a,b)** multiple nodular and cystic lucencies are seen in both lungs, accompanied by bronchiectasis. The lesions are extensively distributed. Six months after the completion of anti-infection therapy: **(c,d)** a reduction in bilateral reticular opacities is noted. The morphology of bronchiectasis has stabilized, with pulmonary consolidations and ground-glass opacities showing signs of subsiding, though residual structural alterations persist.

Readmissions for evaluation occurred on August 12 and September 23, 2024. Physical examination consistently revealed moist rales in the right lower lung. A follow-up chest CT demonstrated multiple nodules and cystic lucencies in both lungs, accompanied by bronchiectasis and peribronchial wall thickening, with extensive distribution of the lesions (Figure 5). Blood tests now revealed bone marrow suppression, with a white blood cell count of 3.44 × 10^9^/L. On September 25, 2024, a follow-up bronchoscopy revealed that the basal segments of the right lower lobe were still obstructed by copious purulent secretions. Bronchoalveolar lavage (BAL) was performed, and the fluid was sent for routine microbiological examination and targeted next-generation sequencing (tNGS). Gram staining of the BAL fluid smear revealed a few Gram-positive cocci without evidence of phagocytosis; acid-fast staining and fungal smear were both negative. Bacterial and fungal cultures of the BAL fluid showed no growth. However, BALF-tNGS detected *Nocardia abscessus* (sequence count: 6,559), confirming persistent infection.

Given the persistent infection, intravenous linezolid was added to oral TMP-SMX and nebulized amikacin. This intensification exacerbated bone marrow suppression: the white blood cell count decreased from 3.44 × 10^9^/L on September 23 to a nadir of 2.59 × 10^9^/L on October 6. Stepwise interventions were initiated, including LiKeJun tablets (a proprietary Chinese medicine) and DiyuShengbai tablets. Given the continued suboptimal therapeutic response, subcutaneous injections of human granulocyte colony-stimulating factor (G-CSF) were added. This aggressive management successfully restored the white blood cell count to 5.58 × 10^9^/L by October 8, and the infection was brought under control, allowing for discharge.

During the subsequent long-term follow-up management, myelosuppression persisted. On November 17, 2024, a follow-up sputum culture newly isolated *Enterobacter cloacae*. Antimicrobial susceptibility testing revealed that the strain was resistant to trimethoprim-sulfamethoxazole, but susceptible to aminoglycosides, third- and fourth-generation cephalosporins, carbapenems, and fluoroquinolones. The treatment strategy was adjusted to continue intravenous linezolid combined with nebulized amikacin, while maintaining oral TMP-SMX. On December 26, 2024, the patient underwent a repeat bronchoscopy, and BALF was sent for tNGS. The results returned positive for *Nocardia abscessus* (sequence count: 3), along with low sequence counts of *Klebsiella* spp. (218) and cytomegalovirus (434). These findings were considered to represent low-level colonization or background flora, and given the patient’s clinical presentation, no specific intervention was undertaken. Concurrent routine microbiological examinations of sputum and BALF, including acid-fast staining, bacterial culture, and fungal culture, all tested negative for pathogens.

**Figure 6:**
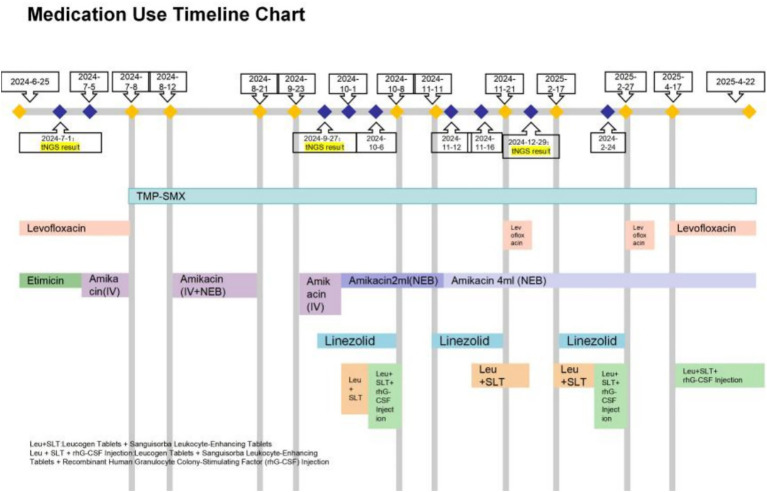
Medication chart for Case 3.

Following a total of 10 months of individualized combination antimicrobial therapy, the patient’s clinical symptoms resolved completely. All anti-infective medications were discontinued on April 22, 2025. Six months after treatment discontinuation (November 2025), follow-up evaluation demonstrated further reduction of bilateral reticular opacities, stable bronchiectasis, and partial regression of pulmonary consolidations and ground-glass opacities, with only residual structural changes remaining (Figure 5). The medication timeline for Case 3 is detailed in Figure 6.

## 3 Literature review

Using “pulmonary nocardiosis” as the keyword, we systematically searched English case reports published in the PubMed database between January 2015 and September 2025. After screening, 109 relevant articles were included, and clinical data from a total of 119 patients were integrated and analyzed. The summarized clinical characteristics are presented in Supplementary Table 1.

Among the 119 patients, the median age was 57 years, with 66 males (55.5%) and 53 females (44.5%). The most common comorbidities were chronic lung diseases (including COPD, bronchiectasis, asthma, pulmonary fibrosis, lung cancer, etc.), accounting for 47 cases (39.5%), followed by immunosuppression-related conditions (including solid organ transplantation, HIV, rheumatoid arthritis, immunosuppressive states, etc.), also with 47 cases (39.5%). Diabetes was present in 19 cases (16.0%).

Chest CT findings were diverse, with consolidation (57 cases, 47.9%) and nodules (49 cases, 41.2%) being the most common manifestations. Cavitary lesions were also frequently observed (36 cases, 30.3%). Other common findings included bronchiectasis (25 cases, 21.1%), ground-glass opacities (13 cases, 10.9%), and pleural effusion (12 cases, 10.1%). The primary specimens for etiological diagnosis were sputum/airway secretions (58 cases, 48.7%) and bronchoalveolar lavage fluid (51 cases, 42.9%). In terms of diagnostic methods, traditional culture remained dominant (85 cases, 71.4%), while molecular diagnostic techniques were widely applied, with 16S rRNA gene sequencing (30 cases, 25.2%), MALDI-TOF MS (17 cases, 14.3%), and metagenomic next-generation sequencing (mNGS) (15 cases, 12.6%) being commonly used (Table 1).

**Table 1:** Findings from the literature analysis.

Category	Details
Total number of cases	119 cases
Gender distribution	Male: 66 people, Female: 53 people
Age	Median age is 57 years old
Prognosis	improved: 105 cases, Death: 7 cases, NA: 7 cases
Comorbidities	Chronic pulmonary diseases (including COPD, bronchiectasis, asthma, pulmonary fibrosis, lung cancer, etc.): *n* = 47 (39.5%); Diabetes: *n* = 19 (16.0%); Immunosuppression-related diseases (including transplantation, HIV, rheumatoid arthritis, immunosuppression, etc.): *n* = 47 (39.5%), of which solid organ transplantation: *n* = 8 (6.7%), HIV: *n* = 8 (6.7%); No clear immunosuppression/other diseases: *n* = 31 (26.1%)
Chest CT	Nodular: *n* = 49(41.2%); Mass-like: *n* = 29(24.4%); Consolidation: *n* = 57(47.9%); Cavitary: *n* = 36(30.3%); Ground-glass opacity (GGO): *n* = 13(10.9%); Reticular: *n* = 5(4.2%); Cystic: *n* = 6(5.0%); Bronchiectasis: *n* = 25(21.1%); Pleural effusion: *n* = 12(10.1%);
Diagnostic specimen types	Bronchoalveolar lavage fluid: *n* = 51(42.9%); Sputum or airway secretions: *n* = 58(48.7%); histopathology: *n* = 17(14.3%); sterile body fluids (including blood, pleural effusion): *n* = 11(9.2%)
Diagnostic method	mNGS: *n* = 15(12.7%); 16S rRNA gene sequencing: *n* = 30(25.2%); MALDI-TOF MS: *n* = 17(14.3%); nanopore sequencing: *n* = 4(3.4%); tNGS: *n* = 3(2.5%); VITEK MS: *n* = 2(1.7%); DNA Sequencing: *n* = 2(1.7%); WGS: *n* = 1(0.8%); culture: *n* = 85(71.4%); PCR: *n* = 2(1.7%); secA1 gene sequencing: *n* = 2(1.7%); standard biochemical tests: *n* = 1(0.8%)
Treatment regimen	Regimens containing trimethoprim-sulfamethoxazole: *n* = 99(83.2%); Regimens containing carbapenem: *n* = 44(36.7%); Regimens containing linezolid: *n* = 25(21.0%); Regimens containing aminoglycoside: *n* = 18(15.1%); Regimens containing Third-generation cephalosporins: *n* = 10(8.4%); Other regimens: *n* = 10(8.4%)
NGS group (number of treated patients: 18 cases)	Death: 1 cases (5.6%), improved: 17 cases (94.4%)
Non-NGS group (number of treated patients: 101 cases)	Death: 6 cases (5.9%), improved: 88 cases (87.1%), NA: 7 cases (6.9%)
Bronchoscopy group (number of treated patients: 76 cases)	Death: 4 cases (5.3%), improved: 69 cases (90.8%), NA: 3 cases (3.9%)
Non-bronchoscopy group (number of treated patients: 43 cases)	Death: 3 cases (7.0%), improved: 36 cases (83.7%), NA: 4 cases (9.3%)

All patients received antimicrobial therapy, with regimens containing trimethoprim-sulfamethoxazole (TMP-SMX) being the most common (99 cases, 83.2%), followed by regimens containing carbapenems (44 cases, 36.7%) and linezolid (25 cases, 21.0%). Bronchoscopy was performed in 76 patients (63.9%) to assist in diagnosis.

The overall prognosis of the patients in this report was favorable, with improvement observed in 105 cases (88.2%) and death in 7 cases (5.9%). Analysis of the association between different diagnostic approaches and mortality showed that patients who underwent NGS testing had a lower mortality rate (1/18, 5.6%) compared to those who did not (6/101, 5.9%). Similarly, patients who underwent bronchoscopic intervention had a lower mortality rate (4/76, 5.2%) than those who did not (3/43, 7.0%).

## 4 Discussion and conclusion

This report, through an integrated analysis of 119 cases of pulmonary nocardiosis, systematically explores the clinical features, diagnosis, and treatment strategies of this disease.

### 4.1 Diagnostic challenges and the value of NGS

The diagnosis of pulmonary nocardiosis relies heavily on etiological identification, as clinical and radiological features are notoriously non-specific. In our review, common presentations such as fever, cough, and sputum (present in all three cases) and imaging findings including nodules (41.2%), consolidation (47.9%), and cavities (30.3%) overlapped considerably with those of tuberculosis, lung cancer, and bacterial pneumonia (4). This diagnostic ambiguity underscores the critical need for precise pathogen detection.

Currently, diagnosis relies on time-consuming bacterial culture methods, which not only delay the initiation of effective treatment but also frequently necessitate frequent empirical adjustments to antibiotic therapy (5). In this report, Matrix-Assisted Laser Desorption/Ionization Time-of-Flight Mass Spectrometry (MALDI-TOF MS) provided identification results for 17 patients. This technique operates on the principle of differences in the mass-to-charge ratios of proteins from different microorganisms. By comparing the protein fingerprint of a single bacterial isolate against a reference database, identification can be achieved (6). 16S rRNA gene sequencing has historically played a key role in the molecular identification of *Nocardia*, compensating for the culture limitations and enabling rapid genus-level and partial species-level identification. It provided crucial diagnostic evidence in 25.2% of cases in this review. However, it has limitations in turnaround time, sensitivity, species-level resolution, and cannot provide antimicrobial resistance data.

NGS offers superior sensitivity, specificity, and faster turnaround (7). It optimizes therapeutic efficacy and establishes itself as a powerful tool for precision diagnosis (8). mNGS was used in 12.7% of cases here. It allows unbiased detection of all potential pathogens in a sample, which is vital for diagnosing mixed or rare infections. However, its widespread clinical adoption is limited by high cost, host nucleic acid interference, and the technical complexities associated with separate DNA and RNA processing (9).

Targeted NGS (tNGS) achieves targeted amplification and high-throughput sequencing of specific genes or pathogens. It combines the specificity of 16S sequencing with the broad detection and resistance-profiling capabilities of mNGS. As used in our cases, tNGS provides a cost-effective, rapid alternative with good detection rates for common pathogens and resistance genes, aligning well with routine clinical needs (10). It has gradually become an important technical method for the precise diagnosis of infectious diseases. This case series reflects this trend: Case 1 confirmed a mixed *Nocardia* and *Moraxella catarrhalis* infection via sputum tNGS; Cases 2 and 3 confirmed a *Nocardia* infection via BALF-tNGS while concurrently detecting *Pseudomonas aeruginosa* and later Aspergillus species.

Despite the significant advancements that NGS technologies have brought to the diagnostic capability for pulmonary nocardiosis, both tNGS and mNGS have certain limitations in clinical application, necessitating careful selection based on specific circumstances. Regarding mNGS, its limitations are primarily manifested in the following aspects: First, the high cost of testing limits its widespread use in resource-limited settings or in scenarios requiring repeat testing. Second, the test results are susceptible to interference from the human nucleic acid background and the respiratory colonizing microbiota, which may lead to difficulties in result interpretation. The limitations of tNGS are mainly reflected in the following areas: First, its detection scope is dependent on preset primers or probes, potentially missing rare or emerging pathogens outside the targeted range. This concern is particularly relevant in the diagnosis of *Nocardia*, a genus with numerous species and continuous discovery of novel strains. Second, although its cost is lower than that of mNGS, the technical threshold for probe design and database update maintenance remains relatively high, indicating room for further optimization.

It should be noted that both techniques are based on DNA sequencing principles and therefore share some common issues. For instance, they cannot distinguish between viable and non-viable organisms, limiting their value in post-treatment assessment. This limitation is a factor that must be considered in clinical decision-making. Therefore, when interpreting NGS results, it is essential to integrate them comprehensively with the patient’s clinical presentation, imaging progression, and other laboratory findings.

### 4.2 The dual role of bronchoscopy in diagnosis and treatment

In our case series, 63.9% of the reviewed patients underwent bronchoscopy. This procedure played a positive role in the diagnosis and management of Cases 2 and 3. Firstly, Bronchoscopy can preliminarily rule out other diseases with clinical manifestations similar to those of *Nocardia* infection under direct visualization, thereby laying the foundation for subsequent targeted etiological testing. Subsequently, it provides high-quality lower respiratory tract specimens for NGS, culture, and susceptibility testing, effectively shortening diagnostic time and avoiding the delays of empirical therapy. Literature indicates that combining routine testing with BALF analysis increases pathogen detection rates to 75.1% (11). Ultimately, for patients with structural lung disease like bronchiectasis, excessive secretions can perpetuate infection and hinder drug penetration. it serves as a therapeutic adjunct by clearing secretions, alleviating obstruction, and improving the local microenvironment for antibiotic penetration.

### 4.3 Underlying diseases and immune status

The majority of patients in this report had one or more underlying conditions, most commonly chronic lung disease, immunosuppression, and diabetes. The resultant cellular immune dysfunction and structural lung damage form the basis for host susceptibility, aligning with the opportunistic nature of *Nocardia* species. The pathogenesis of nocardiosis is closely linked to host immune deficiency, where T-cell-mediated immunity is the primary defense (12). Patients with immune-related diseases often require long-term immunosuppressants, compromising this defense. Literature indicates that structural damage from diseases like COPD and bronchiectasis, epithelial injury from dysbiosis, suppression of alveolar macrophages by inhaled steroids, and frequent antibiotic use all increase susceptibility to opportunistic pathogens (4). Diabetes promotes the proliferation of *Nocardia* by providing carbon sources and impairing neutrophil function (13). Therefore, actively managing comorbidities is fundamental to improving prognosis alongside targeted anti-infective therapy. Given the recalcitrant nature of *Nocardia* infection, treatment typically requires 6–12 months, or continuation for 2–3 months after radiographic improvement, to prevent relapse (14). It is noteworthy that epidemiological studies indicate up to one-third of cases occur in immunocompetent individuals (15), which aligns with the findings of our report.

### 4.4 Treatment strategies and adverse reaction management

Trimethoprim-sulfamethoxazole (TMP-SMX) remains the first-line treatment due to its favorable pharmacokinetics, including high blood and tissue penetration, including into the central nervous system (3). It served as the foundation of therapy for 83.2% of patients in this report.

For severe, disseminated, or TMP-SMX-intolerant/resistant infections, combination therapy with agents like linezolid, carbapenems, or amikacin is standard (2). Multidrug regimens broaden the spectrum through synergy, reduce initial failure risk when susceptibility is unknown (16), and provide a broader therapeutic window for patients with severe conditions or high-level immunosuppression, effectively preventing mixed infections and the development of drug resistance (17).

However, prolonged and combination therapy increases adverse reactions. Patient 3 developed leukopenia during long-term treatment, underscoring the need for systematic blood monitoring and timely intervention. In addition, patients in this group exhibited variable tolerance to TMP-SMX, ranging from gastrointestinal intolerance in Case 1 to systemic reactions in Case 2. This finding emphasizes the importance of flexibly switching medications based on drug susceptibility results and individual patient responses in clinical practice.

### 4.5 Strengths and limitations of the report

A major strength of this case series lies in the combination of detailed case reports with a systematic literature review. The three cases illustrate the practical application of tNGS in the diagnosis, treatment adjustment, and follow-up of complex pulmonary *Nocardia* infections, providing clinical insights for management. Furthermore, the integrated analysis of 119 published cases from the past decade provided valuable insights into the epidemiological characteristics and current diagnostic and therapeutic landscape of this disease.

However, this report has certain limitations. First, its retrospective design and the nature of aggregating data from published case reports may introduce inherent biases. Second, considerable heterogeneity existed across the original studies regarding treatment regimens, follow-up durations, and outcome definitions, which precluded more in-depth analysis.

## 5 Conclusion

In conclusion, the diagnosis of pulmonary nocardiosis remains heavily dependent on etiological identification, given its non-specific clinical presentation and radiological findings. Our findings suggest that a diagnostic approach incorporating bronchoscopy and NGS, coupled with susceptibility-guided and dynamically adjusted combination therapy, may be associated with better clinical outcomes in pulmonary nocardiosis. This association, reflected in the lower mortality trends among patients receiving these interventions in our pooled analysis, warrants confirmation in prospective studies. Furthermore, during prolonged treatment, close monitoring for and timely intervention in adverse drug reactions are also key to successful management.

Based on our findings, the strategic selection between tNGS and mNGS in routine clinical practice should be guided by specimen quality, the suspected pathogen profile, and available medical resources. TNGS is more suitable for targeted rapid screening and routine diagnosis. Specifically, it demonstrates favorable clinical utility in the following scenarios: for relatively stable patients in general wards where the clinical suspicion is well-defined, primarily requiring differentiation among common pathogens such as *Nocardia* spp., mycobacteria, and fungi; in settings with limited healthcare resources or when patients face financial constraints that preclude mNGS testing. Conversely, mNGS is more appropriate for comprehensive pathogen exploration in critically ill patients with suspected rare or complex infections, particularly in immunocompromised hosts. It is indicated for patients with severe and rapidly progressive disease requiring urgent pathogen identification to guide targeted therapy; for diagnostically challenging cases where conventional microbiological tests have repeatedly yielded negative results, yet clinical suspicion for *Nocardia* or other rare pathogens remains high. Importantly, the choice between tNGS and mNGS should be viewed as a dynamic process rather than a static rule. For instance, if initial tNGS is negative in a patient with high clinical suspicion, escalation to mNGS should be strongly considered. Ultimately, further studies are needed to establish evidence-based algorithms.

## Data Availability

The original contributions presented in the study are included in the article/Supplementary material, further inquiries can be directed to the corresponding author.
