# Supplementary material for: Case Report: Pulmonary nocardiosis: three case reports and literature review
Source: Front Med (Lausanne). 2026 Mar 18;13:1789986. doi: 10.3389/fmed.2026.1789986 (PMC13038990; doi:10.3389/fmed.2026.1789986)
Supplement: Supplementary file 1 [file Table_1.docx]

Table 1 Clinical characteristics of 119 patients with pulmonary nocardiosis.

| Case Source | **Age (years)** | **Gender** | **Comorbidities** | **Chest CT** | **Diagnostic Specimen** | **Diagnostic Method** | **Nocardia Species** | **Treatment Regimen** | **Bronchoscopy Used** | **Prognosis** |
| --- | --- | --- | --- | --- | --- | --- | --- | --- | --- | --- |
| 1(1) | 70 | M | Liver transplantation+Diabetes+COPD+Chronic kidney disease | multiple scattered bilateral lung nodules | BALF | MALDI-TOF MS+16S rRNA gene sequencing | Nocardia exalbida | IMP-cilastatin+TMP-SMX | yes | improved |
| 2(2) | 54 | F | Diabetes+Hypothyroidism | Bilateral pulmonary nodules | Lung biopsy | acid-fast staining | NA | TMP-SMX | no | improved |
| 3(3) | 3 | M | Chronic lung disease+Chiari II malformation+Spinal dysgenesis+Hydrocephalus+Vesicoureteral reflux | consolidations+ground-glass opacities+pneumatoceles | BALF | culture | NA | TMP-SMX+caspofungin | yes | death |
| 4(4) | 54 | M | NA | left pyopneumothorax+right peribronchovascular micronodules | Pleural fluid | culture | NA | Amoxicillin-clavulanic acid | no | improved |
| 5(5) | 60-70 | M | COPD+Benign prostatic hyperplasia+Gastritis+Lung adenocarcinoma | pulmonary inflammatory lesions | Sputum | culture | Nocardia cynaciocortical | TMP-SMX+IMP→TMP-SMX | no | NA |
| 6(6) | 68 | M | Idiopathic pulmonary fibrosis+Lung transplantation | left lung scattered ground-glass opacities +right lung fibrosis with honeycombing. | BALF | mNGS | Nocardia farcinica | LZD→TMP-SMX | yes | improved |
| 7(7) | 79 | F | COPD | patchy+nodular+soft tissue masses | BALF | MALDI-TOF MS+16S rRNA gene sequencing | Nocardia farcinica | TMP-SMX + Isoniazid+Rifampin+Pyrazinamide+Ethambutol | yes | improved |
| 8(8) | 45 | M | Kidney transplantation | nodules+treak shadows and fiber lesions+right pleural effusion | Pleural fluid+Blood | mNGS | Nocardia farcinica | TMP-SMX+MINO | no | improved |
| 9(9) | 53 | M | NA | bilateral plaques and nodules+central cavities+bronchiectasis | BALF | mNGS | Nocardia terpenica | TMP-SMX | yes | improved |
| 10(10) | 16 | F | NA | patchy lesions | BALF | mNGS+WGS+MALDI-TOF MS | Nocardia gipuzkoensis | IMP-cilastatin→LZD+Moxifloxacin | yes | improved |
| 11(11) | 78 | M | NA | patchy hyperdense opacities+ground-glass opacities | Pus | mNGS | Nocardia otitidiscaviarum | TMP-SMX + Meropenem | yes | improved |
| 12(12) | 55 | M | Cerebral infarction | miliary nodules+pleural effusion+occupying lesions in the right lower lobe | Sputum | culture | Nocardia otitidiscaviarum | TMP-SMX+Doxycycline | no | improved |
| 13(13) | 61 | M | NA | Mass with spiculated margins in the left upper lobe | Lung biopsy | acid-fast staining | NA | IMP + TMP-SMX | no | improved |
| 14(14) | 48 | M | Seizure disorder+HIV | multiple heterogeneously enhancing lesions | BALF | culture | NA | Antibiotics (specific drug name not listed) | yes | improved |
| 15(15) | 58 | F | Little’s disease | bronchiectasis in the RML+centrilobular nodules+condensed infiltrates | Bronchial secretions | culture | Nocardia cyriacigeorgica | TMP-SMX + Doxycycline→TMP-SMX | yes | improved |
| 16(16) | 56 | M | Latent tuberculosis | cavitary mass+ mild patchy infiltrate seen around the mass | Lung biopsy | acid-fast staining | NA | Meropenem + TMP-SMX→ Amoxicillin-clavulanate + TMP-SMX | yes | improved |
| 17(17) | 75 | M | COPD+Obesity | pulmonary nodules+right basal consolidation+pleural effusion | BALF | culture | Nocardia cynicotecorica | TMP-SMX | yes | improved |
| 18(18) | 72 | M | Diabetes | diffuse heterogeneous opacities | ETA | culture | NA | IMP+LZD→LZD + MINO | no | improved |
| 19(19) | 55 | F | CD4+ T cell deficiency | patchy+nodular+strip-shaped | BALF | MALDI-TOF MS | Nocardia cyriacigeorgica | TMP-SMX | yes | improved |
| 20(20) | 61 | F | Rheumatoid arthritis | nodular lesion | Sputum | 16S rRNA gene sequencing | Nocardia veterana | MINO | no | improved |
| 21(21) | 52 | F | NA | multiple nodules | Lung biopsy+BALF | mNGS | Nocardia farcinica | Piperacillin sodium/sulbactam sodium + Voriconazole→voriconazole | yes | improved |
| 22(22) | 70 | F | NA | diffuse nodules+small cavity | Sputum+BALF | MALDI-TOF MS+16S rRNA gene sequencing | Nocardia sputorum | TMP-SMX→MINO | yes | improved |
| 23(23) | 52 | F | hypothyroidism | ill-defined nodules+consolidation+bronchial wall thickening | BALF | 16S rRNA gene sequencing | Nocardia blacklokiae | Amoxicillin-clavunate+clarithromycin→Amoxicillin-clavunate | yes | improved |
| 24(24) | 70 | F | MAC-PD+hyperlipidaemia+dyspepsia +hypothyroidism. | cavities+bronchiectasis+infiltrative shadows+diffuse nodular | BALF | culture | NA | TMP-SMX→MINO→Levofloxacin | yes | improved |
| 25(25) | 46 | F | Idiopathic CD4 lymphocytopenia | multiple thick-walled cavitary nodules | Sputum+BALF | mNGS | Nocardia otitidiscaviarum | TMP-SMX+AMK→TMP-SMX | yes | improved |
| 26(26) | 75 | M | Diabetes+COPD | lobar consolidation +cavitation | Sputum | 16S rRNA gene sequencing | Nocardia amamiensis | TMP-SMX+IMP | no | improved |
| 27(27) | 69 | M | hypertension+asthma+coronary artery disease+kidney transplant | left lower lobe consolidation | Blood | culture | Nocardia transvalensis | IMP-cilastatin→LZD+Moxifloxacin | no | improved |
| 28(28) | 46 | F | asthma | nodular opacities+peribronchial thickening | BALF | culture | Nocardia nova complex | TMP-SMX | yes | improved |
| 29(29) | 35 | F | AIDS | heterogeneous mass+atelectasis of the RUL | Sputum | MALDI-TOF MS | Nocardia beijingensis | TMP-SMX→Ceftriaxone+AMK→TMP-SMX | no | improved |
| 30(30) | 60 | F | dyslipidaemia | a cavity+infiltration | BALF | 16S rRNA gene sequencing | Nocardia mexicana | BIPM→LZD+AMK→BIPM+AMK→LZD+AMK→MINO+IMP、AMK(or LZD) | yes | improved |
| 31(31) | 67 | M | NA | mass+ground-glass opacity | BALF | mNGS | Nocardia otitidiscaviarum | TMP-SMX | yes | improved |
| 32(32) | 34 | F | AIDS | NA | BALF+Sputum | mNGS+MALDI-TOF MS | Nocardia cyriacigeorgica | TMP-SMX+LZD | yes | improved |
| 33(33) | 67 | M | NA | multiple nodules+mass shadow | BALF | nanopore sequencing | Nocardia otitidiscaviarum | IMP-cilastatin+LZD+TMP-SMX→LZD | yes | improved |
| 34(34) | 52 | F | immune thrombocytopenia | Hyperdense shadow in the right lung+solid nodule in the left lung | BALF | mNGS | Nocardia aobensis | TMP-SMX+IMP→TMP-SMX and LZD→Doxycycline | yes | improved |
| 35(35) | 37 | F | pulmonary sequestration | honeycomb lesion+an enhanced vascular shadow in the descending aorta | BALF | MALDI-TOF MS | Nocardia cyriacigeorgica | TMP-SMX+AMK | yes | improved |
| 36(36) | 69 | M | chronic bronchiectasis+ IgA nephropathy | pleural effusion+left atelectasis | BALF | VITEK MS | Nocardia otitidiscaviarum | TMP-SMX+IMP→TMP-SMX + LZD | yes | improved |
| 37(37) | 8 | F | Neuromyelitis Optica Spectrum Disorders | Left-sided empyema + pericardial effusion | Pleural fluid+BALF | mNGS | Nocardia farcinica | TMP-SMX+AMK+LZD | yes | improved |
| 38(38) | 55 | F | NA | irregular soft tissue density shadow | Lung biopsy | mNGS | Nocardia farcinica | TMP-SMX | yes | improved |
| 39(39) | 54 | M | Pemphigus foliaceus+Diabetes mellitus | thick-walled cavities+fungal ball’ like inclusions | Sputum+BALF | rDNA Sequencing | Nocardia brasiliensis | Ceftriaxone+AMK+TMP-SMX→TMP-SMX | yes | improved |
| 40(40) | 74 | F | COPD | large infiltrating mass on the left+nodules in the right | Sputum | culture | NA | TMP-SMX | no | improved |
| 41(41) | 52 | M | HIV+cervical myelopathy+Diabetes mellitus+Hypertension | cavitary lesions | Sputum+BALF | 16S rRNA gene sequencing | Nocardia caishijiensis | AMK+TMP-SMX→TMP-SMX | yes | improved |
| 42(42) | 46 | F | COPD+Old Tuberculosis | cavitary consolidations+micro-nodules | Sputum+BALF | culture | NA | TMP-SMX | yes | improved |
| 43(43) | 69 | F | Resected right breast cancer | cavitary lung lesion+ nodules+consolidations | BALF | 16S rRNA gene sequencing | Nocardia pneumoniae | IMP→Faropenem | yes | improved |
| 44(44) | 49 | M | NA | right upper lobe and left lower lobe consolidation. | BALF | culture | Nocardia otitidiscaviarum | TMP-SMX+AMK | yes | improved |
| 45(45) | 37 | M | HIV | pleural-based mass with several small air foci | Sputum | culture | Nocardia farcinica | TMP-SMX | no | NA |
| 46(46) | 86 | M | Diabetes+Hypertension+atrial fibrillation+Myeloma+COVID-19 | cavitated condensations+Ground glass pattern+distal bronchial thickening | Sputum | culture | NA | IMP→MINO | no | improved |
| 47(47) | 61 | F | NA | bronchiectasis+pulmonary inflammation | BALF | MALDI-TOF MS | Nocardia wallacei | LZD | yes | improved |
| 48(48) | 62 | F | Non-small cell lung cancer+ diabetes+Cardiovascular disease | ground-glass nodules+necrotic consolidation mass | Sputum | culture | Nocardia cyriacigeorgica | Meropenem+TMP-SMX | no | improved |
| 49(49) | 55 | M | multiple myeloma | ground glass+nodular opacities | BALF | 16S rRNA gene sequencing | Nocardia ignorata | TMP-SMX + IMP→TMP-SMX | yes | improved |
| 50(50) | 47 | M | NA | mass+several discrete complex gas and fluid collections | Lung biopsy | culture | Nocardia beijingensis | TMP-SMX+Doxycycline→Doxycycline | yes | improved |
| 51(51) | 55 | M | hypertension+ischemic heart disease+chronic kidney disease | NA | Pleural fluid | VITEK MS | Nocardia otitidiscaviarum | Meropenem + TMP-SMX | no | death |
| 52(52) | 52 | M | diabetes mellitus+ bronchiectasis+multisystem sarcoidosis+sarcoidosis | lung nodules | Sputum | MALDI-TOF MS | Nocardia pseudobrasiliensis | TMP-SMX + IMP + LZD→LZD +ciprofloxacin→TMP-SMX+ciprofloxacin | no | improved |
| 53(53) | 37 | M | NA | multiloculated collection+compression atelectasis | Lung tissue | nanopore sequencing | NA | IMP + TMP-SMX | no | improved |
| 54(54) | 79 | M | asthma+lung cancer | contrast-enhancing effect around the right main bronchus+nodules | Sputum | MALDI-TOF MS | Nocardia araoensis | TMP-SMX | yes | improved |
| 55(55) | 41 | F | asthma+hepatitis B+COPD | bronchiectasis+superimposed infection | BALF + Biood | MALDI-TOF MS+16S rRNA gene sequencing | Nocardia amamiensis | Ceftriaxone + TMP-SMX→TMP-SMX | yes | improved |
| 56(56) | 61 | M | diabetes mellitus | infiltrative shadows+nodular+ground-glass opacity+bronchiectasis | Bronchial wash | 16S rRNA gene sequencing | Nocardia cyriacigeorgica | TMP-SMX | yes | improved |
| 57(57) | 56 | M | diabetes mellitus+ fibrotic NSIP | subpleural honeycombing+diffuse ground-glass opacities+traction bronchiectasis | Sputum | culture | Nocardia farcinica | Meropenem→AMK+TMP-SMX→TMP-SMX | no | improved |
| 58(58) | 70 | M | NA | consolidation with internal breakdown+ pleural effusion | Sputum | culture | NA | TMP-SMX + Meropenem → LZD + TMP-SMX | no | improved |
| 59(59) | 76 | M | rheumatoid arthritis | diffuse infiltration+bronchiectasis | BALF | culture | Nocardia astrousis | cefoperazone sodium/sulbactam sodium+TMP-SMX+voriconazole→TMP-SMX+voriconazole | yes | improved |
| 60(60) | 58 | M | nephrotic syndrome | effusion+multiple small nodular+strip-like dense shadows | BALF | mNGS | Nocardia brasiliensis | TMP-SMX+human albumin | yes | improved |
| 61(61) | 73 | F | diabetes mellitus+hypertension+cerebral infarction | diffuse nodules+mass shadow | BALF | mNGS | Nocardia otitidiscaviarum | TMP-SMX+IMP-cilastatin→TMP-SMX | yes | improved |
| 62(62) | 69 | F | renal transplantation | well-defined nodules+cavitary masses+ ground-glass opacities | BALF | nanopore sequencing+16S rRNA gene sequencing | Nocardia elegans | TMP-SMX | yes | improved |
| 63(63) | 53 | M | asthma+diabetes+atopic rhinitis+nasal polyps | bibasilar bronchiectasis+ pulmonary nodules+ground-glass opacity | BALF+bronchial wash | Partial 16S rRNA gene sequencing | Nocardia nova | TMP-SMX→Ceftriaxone→Clarithromycin | yes | improved |
| 64(64) | 45 | M | pulmonary alveolar proteinosis | local consolidation | Lung biopsy | nanopore sequencing | NA | Sulfamethoxazole | yes | improved |
| 65(65) | 53 | F | autoimmune cirrhosis | multiple thick-walled cavities | Sputum | MALDI-TOF MS | Nocardia cyriacigeorgica | TMP-SMX+voriconazole+IMP+caspofunging | no | NA |
| 66(66) | 86 | F | colorectal cancer+diabetes mellitus | tumour-like shadows+ground-glass opacity+cavitary lesions | Bronchial wash | 16S rRNA gene sequencing | Nocardia nova | TMP-SMX+IMP-cilastatin→TMP-SMX→MINO | yes | death |
| 67(67) | 65 | M | Chronic inflammatory demyelinating polyneuropathy | consolidation shadows+nodular high-density shadows+cavities | Sputum+BALF | mNGS | Nocardia gelsenkirchen | TMP-SMX+Meropenem+LZD | yes | death |
| 68(68) | 71 | M | Myasthenia gravis+Diabetes+disease | consolidation in the left lung | Sputum | 16S rRNA gene sequencing | Nocardia cyriacigeorgica | TMP-SMX | no | improved |
| 69(69) | 68 | M | Chronic Obstructive Pulmonary Disease+Diabetes | Mass shadow+bilateral pulmonary nodules+cavity | Sputum+BALF | 16S rRNA gene sequencing+MALDI-TOF MS | Nocardia otitidiscaviarum | TMP-SMX+Moxifloxacin | yes | improved |
| 70 (case 2) | 54 | F | Bronchiectasis+COPD | Consolidation+thickening of the lung marking | BALF | tNGS | NA | LZD+Levofloxacin+AMK | yes | improved |
| 71(case 1) | 68 | F | Hypertension+Bronchiectasis | Consolidation+thickening of the lung marking | Sputum | tNGS | Nocardia asiatica | TMP-SMX+Ceftazidime | no | improved |
| 72(case 3) | 65 | F | Bronchiectasis | Multiple nodules+honeycomb - shaped high - density shadow+bronchiectasis | BALF | tNGS | Nocardia abscessus | TMP-SMX + AMK + LZD + Levofloxacin | yes | improved |
| 73(70) | 55 | F | NA | mass+cavitary lesion | BALF | PCR+culture | Nocardia beijingensis | TMP-SMX+Ceftriaxone | yes | improved |
| 74(71) | 50 | F | Breathlessness | necrotizing consolidation+loculated hydropneumothorax | Sputum+BALF | culture | NA | TMP-SMX+AMK→TMP-SMX+ LZD→Doxycycline | yes | improved |
| 75(71) | 72 | M | breathlessness( oral steroids for over five years ) | necrotizing consolidation+cavitating+nodules | BALF | culture | NA | TMP-SMX+ LZD→Doxycycline+ LZD | yes | death |
| 76(71) | 42 | M | breathlessness( oral steroids for over three years ) | consolidations+air bronchograms | BALF | culture | NA | TMP-SMX+Ceftriaxone→TMP-SMX+ | yes | improved |
| 77(72) | 66 | M | Ischemic cardiomyopathy+Heart transplant+COPD+hypersensitivity lung disease | multiple areas of consolidation with cavitation | Lung biopsy | 16S rRNA gene sequencing+ secA1gene sequencing+MALDI-TOF MS+culture | Nocardia thailandica | TMP-SMX+Meropenem→MINO+Meropenem→Minocyclin | no | improved |
| 78(73) | 75 | M | COPD+chronic catarrhal bronchitis+benign prostatic hypertrophy | Multiple nodular opacities+small and regular central cavitations+Multiple focal points of ‘tree in bud’ | Sputum | 16S rRNA gene sequencing+culture | Nocardia cyriacigeorgica | TMP-SMX | no | improved |
| 79(74) | 77 | M | COPD+right pachypleuritis | NA | Sputum | 16S rRNA gene sequencing+culture | Nocardia fusca | ciprofloxacin | no | NA |
| 80(75) | 77 | M | Myasthenia gravis | cavitary pneumonia+extensive subcutaneous emphysema and pneumomediastinum | BALF | DNA sequencing+culture | Nocardia cyriacigeorgica | TMP-SMX+Meropenem+ LZD→TMP-SMX | yes | improved |
| 81(76) | 67 | M | Renal transplant,+Ischemic heart disease+hypertension+BK nephropathy | spiculated left hilar mass | Pleural fluid+Sputum | culture | Nocardia farcinica | Meropenem+TMP-SMX→TMP-SMX | yes | improved |
| 82(77) | 48 | M | Adult-onset Still‘s disease | nodules+cavitation+bilateral multifocal ground-glass opacities | Lung biopsy+Sputum | culture | Nocardia asiatica | TMP-SMX+IMP+AMK→TMP-SMX+MINO | no | improved |
| 83(78) | 46 | M | Idiopathic CD4 T-lymphocytopenia | a 6.2 × 5.7-cm² mass with some cavitation+air bronchograms | bronchial washings | culture | Nocardia abscessus | IMP+TMP-SMX→Meropenem+TMP-SMX | yes | NA |
| 84(79) | 70 | M | lung cancer+adiation pneumonitis | infiltrative shadows with air bronchograms in the right middle lobe+opacities in the hilum of the right lung | BALF+sputum | 16S rRNA gene sequencing | Nocardia exalbida | Doripenem+TMP-SMX | yes | improved |
| 85(80) | 51 | M | NA | fibrotic changes | BALF | culture | NA | TMP-SMX | yes | improved |
| 86(81) | 60 | F | atopy+ alpha-1 antitrypsin deficiency+bronchiectasis | extensive emphysema+bronchiectasis+multiple indistinct pulmonary opacities | Sputum | 16S rRNA gene sequencing | Nocardia cyriacigeorgica | TMP-SMX | no | improved |
| 87(82) | 61 | F | hyperlipidaemia+bronchiectasis | scattered nodules+bronchiectasis | BALF | 16S rRNA gene sequencing | Nocardia mexicana | TMP-SMX+MINO→Biapenem→AMK+ LZD | yes | improved |
| 88(83) | 41 | M | HIV | pyopneumothorax | Pleural fluid | culture | NA | TMP-SMX+AMK→TMP-SMX | no | improved |
| 89(84) | 58 | M | hepatitis B virus carrier | nodules+masses+patchy consolidations+bilateral pleural effusion | Sputum | mass spectroscopy | Nocardia otitidiscaviarum | TMP-SMX+AMK+IMP | no | death |
| 90(85) | 40 | M | bronchiectasis+pulmonary tuberculosis | bilateral midzone and lower zone consolidation | Sputum | 16S rRNA gene sequencing | Nocardia cyriacigeorgica | IMP+TMP-SMX→TMP-SMX | no | improved |
| 91(85) | 28 | F | bronchiectasis | bilateral mid and lower zone consolidation | Sputum | 16S rRNA gene sequencing | Nocardia beijingensis | TMP-SMX | no | improved |
| 92(86) | 43 | F | glomerulonephritis | multiple cavitary lesions | BALF | 16S rRNA gene sequencing+secA1gene sequencing+culture | Nocardia amamiensis | IMP-cilastatin+TMP-SMX→TMP-SMX | yes | improved |
| 93(87) | 35 | F | asthma | new consolidation | endobronchial mucus | culture | NA | TMP-SMX | yes | improved |
| 94(88) | 71 | M | Multiple Myeloma | a mass-like consolidation+bilateral airspace consolidations | respiratory tract sample | culture | Nocardia abscessus | TMP-SMX+Meropenem→TMP-SMX+MINO→tigecycline+Meropenem | yes | improved |
| 95(89) | 76 | M | NA | a 45-mm mass | Lung biopsy | culture | Nocardia exalbida | Meropenem+TMP-SMX→TMP-SMX+Levofloxacin | yes | improved |
| 96(90) | 40 | F | diabetes+endogenous hypercortisolaemia | two nodules in the right middle lobe, one of which cavitated | Lung biopsy | culture | NA | TMP-SMX | yes | NA |
| 97(91) | 57 | F | diabetes+pancreas transplant+renal disease | bilateral bronchovascular micronodules+focal consolidative opacity | Lung biopsy | 16S rRNA gene sequencing | Nocardia farcinica | TMP-SMX+Amoxicillin/Clavulanate+Moxifloxacin | yes | improved |
| 98(92) | 64 | F | pulmonary tuberculosis | nodular dense lesion | Sputum+BALF | 16S rRNA gene sequencing | Nocardia asiatica | TMP-SMX | yes | improved |
| 99(93) | 64 | M | AIDS+chronic hepatitis C+esophageal candidiasis+herpes simplex | thick wall cavitary lesion+diffuse ground-glass opacities | blood+bronchial aspirate | culture | Nocardia farcinica | TMP-SMX+ceftriaxone→TMP-SMX+Moxifloxacin | yes | improved |
| 100(94) | 65 | M | COPD+pulmonary tuberculosis | diffuse bilateral airspace opacities+right upper lobe fibrosis | Sputum | culture+standard biochemical tests. | Nocardia brasiliensis | TMP-SMX→IMP | no | death |
| 101(95) | 72 | F | bronchial asthma | irregularly shaped solid opacity+cavitary mass+bronchiectasis | Sputum | 16S rRNA gene sequencing | Nocardia otitidiscaviarum | TMP-SMX+ceftriaxone→TMP-SMX+MINO→MINO→Levofloxacin | no | improved |
| 102(96) | 9 | F | Cystic fibrosis,pancreatic insufficiency,allergic bronchopulmonary aspergillosis | consolidation+multifocal pneumonia | Sputum | culture | Nocardia transvalensis | TMP-SMX+ LZD→TMP-SMX + Amoxicillin-clavulanate→TMP-SMX | yes | improved |
| 103(97) | 49 | M | AIDS | cavitary lesion+ nodules | Lung biopsy | culture | NA | NA | yes | NA |
| 104(98) | 50 | F | multiple sclerosis | bilateral infiltrates | Sputum | culture | Nocardia beijingensis | Meropenem | no | improved |
| 105(99) | 57 | M | NA | nodular lesions | BALF | culture+16S rRNA gene sequencing | Nocardia mexicana | LZD+AMK | yes | improved |
| 106(100) | 71 | F | COPD | severe centrilobular emphysema | Sputum | culture | Nocardia cyriacigeorgica | TMP-SMX | no | improved |
| 107(100) | 68 | F | breast cancer | bronchiectasis+tree-in-bud opacities | Sputum | culture | Nocardia cyriacigeorgica | TMP-SMX→Tobramycin+ LZD | no | improved |
| 108(100) | 75 | F | COPD+chronic MAC colonization with bronchiectasis | bronchiectasis+tree-in-bud opacities | Sputum | culture | Nocardia cyriacigeorgica | TMP-SMX | yes | improved |
| 109(100) | 77 | F | bronchiectasis+MAC colonization presented+lung adenocarcinoma | stable bilateral nodules+new consolidation | Sputum | culture | Nocardia nova | Observation (without treatment) | no | improved |
| 110(101) | 36 | F | NA | mass lesion | Lung biopsy | culture+acid-fast staining | NA | TMP-SMX→IMP-cilastatin → LZD | no | improved |
| 111(102) | 17 | M | NA | cavitary lesion | Lung biopsy | PCR | NA | IMP + TMP-SMX →TMP-SMX | no | improved |
| 112(103) | 82 | F | bronchiectasis | small centrilobular nodules+ectatic bronchi | Sputum+Bronchial washing | culture+16S rRNA gene sequencing | Nocardia beijingensis | TMP-SMX+MINO → TMP-SMX | yes | improved |
| 113(104) | 65 | M | NA | infiltrative lesions | Sputum | MALDI-TOF+16S rRNA gene sequencing | Nocardia otitidiscaviarum | TMP-SMX | no | improved |
| 114(105) | 81 | F | B-cells non-Hodgkin lymphoma+diabetes mellitus+bronchiectasis | small centrilobular nodules+consolidation | Tracheal aspirate+Bronchial aspirate | culture+MALDI-TOF MS | Nocardia cyriacigeorgica | TMP-SMX+Meropenem | yes | improved |
| 115(106) | 16 | M | Crohn’s disease)infliximab) | nodule+central area of necrosis | Lung biopsy | culture | Nocardia pseudobrasilliensis | TMP-SMX | no | improved |
| 116(107) | 32 | M | Crohn’s disease)infliximab) | pulmonary nodules+a cavernous mass | Sputum | culture | NA | TMP-SMX | yes | improved |
| 117(108) | 55 | F | Allergic bronchopulmonary aspergillosis | central bronchiectatic changes+patchy shadows | Sputum | MALDI-TOF MS+16S rRNA gene sequencing | Nocardia cyriacigeorgica | TMP-SMX+Meropenem → TMP-SMX | no | improved |
| 118(109) | 52 | M | immune-associated hematocytopenia | sheet shadow contained cavitation | Sputum | culture | Nocardia asteroides | TMP-SMX+voriconazole→cefoperazone-sulbactam+TMP-SMX→IMP and cilastatin +TMP-SMX→cefdinir+TMP-SMX | yes | improved |
| 119(109) | 37 | M | NA | central occupying lesions+obstructive pneumonia | Pleural fluid | culture | NA | TMP-SMX+cefminox →Ceftriaxone+TMP-SMX | yes | improved |

Abbreviations:BIPM: Biapenem; LZD: Linezolid ;AMK: Amikacin; MINO: Minocycline; IMP: Imipenem; TMP-SMX: Trimethoprim-sulfamethoxazole; BALF: Bronchoalveolar lavage fluid;mNGS: Metagenomic next-generation sequencing;tNGS: Targeted next-generation sequencing; MALDI-TOF MS: Matrix-assisted laser desorption/ionization time-of-flight mass spectrometry; WGS: Whole-genome sequencing; ETA: Endotracheal aspirate; NA: Not available/Not identified ;→: Indicates change in treatment regimen;+: Indicates combination therapy

### **References**

1. Adachi-Katayama M, Hashimoto H, Hagiwara S, Yamashita M, Mihara Y, Kanematsu A, Otani A, Wakimoto Y, Oyabu T, Jubishi D, et al. Pulmonary Nocardiosis Due to Nocardia exalbida Infection Following Living-donor Liver Transplantation. Intern Med (2025) 64:965–969. doi: 10.2169/internalmedicine.4085-24

2. Alhumaidi AA, Aljohani EM, Althakafi WA, Alkinani OM, Albalawi FA. The balance between autoimmunity and infection: a case report of concomitant pathology of pulmonary nocardia and granulomatosis with polyangiitis at initial presentation. European Journal of Case Reports in Internal Medicine (2025) 12: 005756.doi: 10.12890/2025_005756

3. Ali AH, Kamal M, Elyan ME. Invasive Pulmonary Aspergillosis and Nocardia Pneumonia in a Pediatric Patient With Chronic Lung Disease: A Case Report. Case Reports in Pediatrics (2025) 2025:6659641. doi: 10.1155/crpe/6659641

4. Bounoua F, Daoudi N, Aghrouch M, Hanchi AL, Soraa N, Serhane H, Moubachir H. Pleuropulmonary nocardiosis, an unusual radiological presentation: Case report. Radiology Case Reports (2023) 18:2725–2729. doi: 10.1016/j.radcr.2023.04.057

5. Calvo M, Beunza Sola M, Tirapu B, Sarobe Carricas M, Moreno E. Therapeutic drug monitoring of linezolid in a case of pulmonary nocardiosis: a case report. Eur J Hosp Pharm (2025)ejhpharm-2024-004462. doi: 10.1136/ejhpharm-2024-004462

6. Cao L, Sun Y, Chen F. Pulmonary nocardiosis following COVID-19 in a patient with idiopathic pulmonary fibrosis and lung transplantation: a case report. Front Med (2023) 10:1266857. doi: 10.3389/fmed.2023.1266857

7. Chen Y, Hu W. Co-infection with Mycobacterium tuberculosis and Nocardia farcinica in a COPD patient: a case report. BMC Pulm Med (2023) 23:136. doi: 10.1186/s12890-023-02434-3

8. Deng Z-F, Tang Y-J, Yan C-Y, Qin Z-Q, Yu N, Zhong X-B. Pulmonary nocardiosis with bloodstream infection diagnosed by metagenomic next-generation sequencing in a kidney transplant recipient: A case report. World J Clin Cases (2023) 11:1634–1641. doi: 10.12998/wjcc.v11.i7.1634

9. Dong J, Guan W, Hu A, Luo Q. Mild Pulmonary Nocardiosis Caused by Nocardia terpenica in an Immunocompetent Patient. Intern Med (2023) 62:2911–2917. doi: 10.2169/internalmedicine.9740-22

10. Duan Y, Zhang X, Deng W, Wang S, Hu J, Wang X, Li W, Chen B. The first reported pulmonary nocardiosis caused by Nocardia gipuzkoensis resisted to trimethoprim/sulfamethoxazol (TMP-SMZ) in an immunocompetent patient. Journal of Global Antimicrobial Resistance (2024) 37:214–218. doi: 10.1016/j.jgar.2024.02.008

11. Fan N, Fang H, Huang F, Zhou J, Liu P, Li M-J, Ding Y-Y. Metagenome next-generation sequencing plays a key role in the diagnosis and selection of effective antibiotics on the treatment of Nocardia pneumonia: a case report. Front Med (2024) 11:1373319. doi: 10.3389/fmed.2024.1373319

12. Feng Y, Zuo C-L, Shi J-X. Brucella infection combined with Nocardia infection: A case report and literature review. Open Life Sciences (2024) 19:20220815. doi: 10.1515/biol-2022-0815

13. Garcia Rueda JE, García Rueda KY, Bermúdez Flórez AM, Peña Mejía LA, Cardona Palacio A, Castaño Ruiz W. Nocardia in an Immunocompetent Patient Simulating Pulmonary Carcinoma: A Case Report and Literature Review. Cureus (2024)16:e64491. doi: 10.7759/cureus.64491

14. George E, T CM, Baikunje N, Nair N. Hidden Behind a Veil: A Rare Case of Pulmonary Nocardiosis. Cureus (2023)15:e38635. doi: 10.7759/cureus.38635

15. Goel MK, Kumar A, Maitra G, Mehta L. Bleeding track sign during endobronchial ultrasound: A signal for caution. Lung India (2025) 42:380–381. doi: 10.4103/lungindia.lungindia_645_24

16. Gonzalez LM, Venkatesan R, Amador P, Sanivarapu RR, Rangaswamy B. TB or Not TB: Lung Nocardiosis, a Tuberculosis Mimicker. Cureus (2024) doi: 10.7759/cureus.55412

17. González-Jiménez P, Méndez R, Latorre A. Pulmonary Nocardiosis. A case report. Rev Esp Quimioter (2022) 35:114–116. doi: 10.37201/req/s01.24.2022

18. Gupta N, Varma M, Sheshadri S, Saravu K. Pulmonary nocardiosis in an elderly man. BMJ Case Rep (2020) 13:e234090. doi: 10.1136/bcr-2019-234090

19. Hong X, Ji Y-Q, Chen M-Y, Gou X-Y, Ge Y-M. Nocardia cyriacigeorgica infection in a patient with repeated fever and CD4+ T cell deficiency: A case report. World J Clin Cases (2023) 11:1175–1181. doi: 10.12998/wjcc.v11.i5.1175

20. Horino T, Ohnishi H, Komori M, Terada Y. Pulmonary Nocardiosis in a Patient with Rheumatoid Arthritis. Intern Med (2024) 63:2107–2108. doi: 10.2169/internalmedicine.2910-23

21. Huang H-Y, Bu K-P, Liu J-W, Wei J. Overlapping infections of Mycobacterium canariasense and Nocardia farcinica in an immunocompetent patient: A case report. World J Clin Cases (2024) 12:2079–2085. doi: 10.12998/wjcc.v12.i12.2079

22. Irifune S, Ide S, Koga S, Mine K, Sugasaki N, Kosai K, Fukuda M, Yanagihara K, Mukae H. Pulmonary Nocardiosis Caused by Nocardia sputorum Identified via 16S rRNA Gene Sequencing: A Case Report. Cureus (2024) doi: 10.7759/cureus.66137

23. Ito Y, Tanigawa M, Yaguchi T, Toyoshima H, Iwamoto K, Nigi A, Itani H, Kondo S, Tokui T, Sasano H. Pulmonary nocardiosis caused by Nocardia blacklokiae in an immunocompetent patient. Respiratory Medicine Case Reports (2020) 29:101005. doi: 10.1016/j.rmcr.2020.101005

24. Ito Y, Miwa S, Shirai M. Pulmonary nocardiosis following nodular bronchiectatic Mycobacterium avium complex pulmonary disease in an immunocompetent patient. BMJ Case Rep (2023) 16:e256007. doi: 10.1136/bcr-2023-256007

25. Kanagiri T, Meena DS, Kumar D, Midha NK, Kombade S, Yadav T. Recurrent pulmonary nocardiosis due to Nocardia Otitidiscaviarum in a patient with isolated CD4 lymphocytopenia: a case report. BMC Infect Dis (2024) 24:1033. doi: 10.1186/s12879-024-09981-y

26. Kanakan A, Kumar A, Kaur U, Narwade P, Rain Z, Yadav N, Kumar I, Kumar D, Chakrabarti SS. Case Report: Nocardia amamiensis Infection Leading to Worsening of Chronic Obstructive Pulmonary Disease Symptoms in an Elderly Man. The American Journal of Tropical Medicine and Hygiene (2023) 109:1137–1140. doi: 10.4269/ajtmh.23-0284

27. Kapoor R, Adapa S, Vakiti A, Gani IY, Mulloy L, Padala SA. A Rare Case of Drug-Resistant Nocardia transvalensis Infection in a Renal Transplant Patient. Journal of Investigative Medicine High Impact Case Reports (2020) 8:2324709620909243. doi: 10.1177/2324709620909243

28. Khan S, Ignatowicz A, Balaji N, Chew CR, Mihilli A, Patel U. Unremitting Asthma as a Presentation of Pulmonary Nocardiosis: A Case Report. Cureus (2024) 16:e54722. doi: 10.7759/cureus.54722

29. Kiatsuranon P, Suwanpimolkul G. Pulmonary Nocardiosis with Superior Vena Cava Syndrome in a HIV-Infected Patient: a Rare Case Report in the World. Jpn J Infect Dis (2021) 74:151–153. doi: 10.7883/yoken.JJID.2019.470

30. Kogure M, Takase E, Fusamoto A, Sato K, Tsuchihashi Y, Nakanishi H, Ikeda T, Kuchibiro T, Hirai Y, Kanai K. Treatment of refractory localized pulmonary nocardiosis caused by Nocardia mexicana with a combination of medication and surgery. Respirology Case Reports (2023) 11:e01098. doi: 10.1002/rcr2.1098

31. Lai Y, Zhou F, Wang H, He X, Zhang Q, Zhou Y. Application of bronchoalveolar lavage fluid cytomorphology in diagnosing Nocardia otitidiscaviarum: a case report. J Med Case Reports (2024) 18:577. doi: 10.1186/s13256-024-04920-6

32. Li J, Zhou Y, Zou N, Chen M. A Case of Nocardia cyriacigeorgica Infection and Literature Review. Cureus (2025) doi: 10.7759/cureus.87189

33. Li D, Liu Q, Xi X, Huang Z, Zhu C, Ding R, Zhang Q. Case Report: Nanopore Sequencing-Based Detection of Pulmonary Nocardiosis Caused by Nocardia Otitidiscaviarum in an Immunocompetent Patient. IDR (2025) Volume 18:1753–1759. doi: 10.2147/IDR.S507194

34. Liang X, Liu X, Huang Z, Qiu F, Jiang Y, Li C, Deng Z, Wu J. Case report: Metagenomic next-generation sequencing for the diagnosis of rare Nocardia aobensis infection in a patient with immune thrombocytopenia. Front Med (2024) 11:1425655. doi: 10.3389/fmed.2024.1425655

35. Lin J, Wu X-M, Peng M-F. Nocardia cyriacigeorgica infection in a patient with pulmonary sequestration: A case report. WJCC (2021) 9:2367–2372. doi: 10.12998/wjcc.v9.i10.2367

36. Lin Y, Jiang Z-Z, Chi X-Q, Chen J-S, Wen C, Zhang C, Wang Y-Y, Xie G-L. Severe pneumonia caused by Nocardia otitidiscaviarum in a patient with bronchiectasis and IgA nephropathy: a case report. Front Med (2025) 12:1496814. doi: 10.3389/fmed.2025.1496814

37. Liu L, Huang Y, Shu S, Zhou H, Fang F, Liu X. Nocardia farcinica pneumonia complicated by pneumocystis jiroveci infection in children with Neuromyelitis Optica Spectrum Disorders: a case report and literature review. Ital J Pediatr (2024) 50:255. doi: 10.1186/s13052-024-01827-2

38. Liu Q, Li Q, Liu B, Fu T. Pulmonary nocardiosis with hilar mass misdiagnosed as lung cancer: A case report. Medicine (baltimore) (2025) 104:e42524. doi: 10.1097/MD.0000000000042524

39. Luo N, Tan S, Li X, Liu S, Singh S, Chen M, Yang W, He Y, Chen C, Liang M. Pulmonary nocardiosis in a patient with pemphigus foliaceus: case report and literature review. BMC Infect Dis (2021) 21:8. doi: 10.1186/s12879-020-05673-5

40. Mahat JB, Hussien S, Negassa RM, Reddy Y, Ayele GM, Michael MB. Nocardia in an Immunocompetent Host Masquerading As Lung Cancer: A Case Report. Cureus (2022) doi: 10.7759/cureus.27039

41. Malaguez Webber F, Nachiappan A, Lau FD, Costello C, Zane S. Nocardia caishijiensis infection: a case report and review of the literature. BMC Infect Dis (2023) 23:218. doi: 10.1186/s12879-023-08186-z

42. Meena DS, Kumar D, Bohra GK, Garg MK, Yadav P, Sharma A, Abhishek KS, Garg P, Pamnani J. Pulmonary nocardiosis with aspergillosis in a patient with COPD: a rare co-infection. IDCases (2020) 20:e00766. doi: 10.1016/j.idcr.2020.e00766

43. Nakagoshi K, Yaguchi T, Takahashi K, Morizumi S, Nishiyama M, Takahashi Y, Iwamura S, Sumitomo K, Shinohara T. Pulmonary nocardiosis caused by Nocardia pneumoniae mimicking non-tuberculous mycobacterial disease. QJM: An International Journal of Medicine (2022) 115:625–626. doi: 10.1093/qjmed/hcac126

44. Nallamotu S, Reddy MS. Exposing the Masquerade of Nocardia otitidiscaviarum Pneumonia: A Case Report. Cureus (2024) doi: 10.7759/cureus.67849

45. Nso N, Nassar M, Guzman Perez LM, Shaukat T, Trandafirescu T. Localized Pulmonary Nocardia farcinica Infection As the Presenting Symptom of Acquired Immunodeficiency Syndrome. Cureus (2021) doi: 10.7759/cureus.17611

46. Ortiz J, Jover F, Ortiz De La Tabla V, Delgado E. Pulmonary nocardiosis after covid-19 infection: case report and literature review. Rev Esp Quimioter (2023) 36:421–424. doi: 10.37201/req/132.2022

47. Pan W, Zhuo B, Wang S, Long J, Xu W, Chen M, Hong X, Ge Y. First report of Nocardia wallacei infection in an immunocompetent patient in Zhejiang province. Open Life Sciences (2024) 19:20220891. doi: 10.1515/biol-2022-0891

48. Quartermain L, Buchan CA, Kilabuk E, Wheatley-Price P. Pulmonary Nocardiosis in a Non-Small Cell Lung Cancer Patient Being Treated for Pembrolizumab-Associated Pneumonitis. Case Rep Oncol (2024) 17:1222–1228. doi: 10.1159/000541694

49. Rahdar HA, Gharabaghi MA, Bahador A, Shahraki-Zahedani S, Karami-Zarandi M, Mahmoudi S, Feizabadi MM. Pulmonary Nocardia ignorata Infection in Gardener, Iran, 2017. Emerg Infect Dis (2020) 26:610–611. doi: 10.3201/eid2603.180725

50. Raslan R, Bailey P, Sastry S. Nocardia beijingensis lung mass in an immunocompetent adult. BMJ Case Rep (2021) 14:e237454. doi: 10.1136/bcr-2020-237454

51. Srivastava S, Samaddar A, Khan S, Tak V, Bohra GK, Sharma D, Ghosh A, Nag VL. Nocardia otitidiscaviarum causing pulmonary nocardiosis: a case report and its review of the literature. Access Microbiol (2024) 6: 000530.v5.doi: 10.1099/acmi.0.000530.v5

52. Stamos DB, Barajas-Ochoa A, Raybould JE. Nocardia pseudobrasiliensis Co-infection in SARS-CoV-2 Patients. Emerg Infect Dis (2023) 29:696–700. doi: 10.3201/eid2904.221439

53. Sukahri S, Zainudin LD, Hadi MF, Firdaus MA-BM, Hafidz MIA. Recurrent Empyema Thoracic Secondary to Pulmonary Nocardiosis in Immunocompetent Patients. Case Reports in Pulmonology (2020) 2020:1–5. doi: 10.1155/2020/8840920

54. Tajima Y, Tashiro T, Furukawa T, Murata K, Takaki A, Sugahara K, Sakagami A, Inaba M, Marutsuka T, Hirata N. Pulmonary Nocardiosis With Endobronchial Involvement Caused by Nocardia araoensis. CHEST (2024) 165:e1–e4. doi: 10.1016/j.chest.2023.07.067

55. Tan J, Huang B, Luo S, Li R, Hou J, Zhang R, Wang L, Liu Y, Li F, Feng J. A rare case of severe pneumonia combined with septic shock caused by Nocardia amamiensis. Travel Medicine and Infectious Disease (2024) 59:102710. doi: 10.1016/j.tmaid.2024.102710

56. Tsuchiya Y, Nakamura M, Oguri T, Taniyama D, Sasada S. A Case of Asymptomatic Pulmonary Nocardia cyriacigeorgica Infection With Mild Diabetes Mellitus. Cureus (2022) doi: 10.7759/cureus.24023

57. V N, Thangaswamy D. Pulmonary Nocardia farcinica Infection in a Non-immunocompromised Patient With Fibrotic Nonspecific Interstitial Pneumonia (NSIP). Cureus (2025) doi: 10.7759/cureus.89074

58. Vaddepally CR, Prasad VP, Sheshala K, Prakasham S, Maturu VN. Post-COVID acute pulmonary nocardiosis: Another novel post-COVID infection. Lung India (2023) 40:90–91. doi: 10.4103/lungindia.lungindia_288_22

59. Wang L, Liu Y, Li H. Co-infection of Nocardia and Aspergillus fumigatus in a immunosuppressed patient: Case report. Medicine (2024) 103:e37073. doi: 10.1097/MD.0000000000037073

60. Wang Y, He X, Liu S, Li X. Pneumocystis jirovecii and Nocardia pneumonia in a middle-aged male with Nephrotic syndrome: a case report and literature review. BMC Infect Dis (2024) 24:1071. doi: 10.1186/s12879-024-09987-6

61. Wang S, Zhang X, Liu Y, Wang M. Metagenomic next-generation sequencing for diagnosis of severe pneumonia caused by Nocardia otitidiscaviarum. J Infect Dev Ctries (2025) 19:1269–1275. doi: 10.3855/jidc.20869

62. Watanabe C, Kimizuka Y, Fujikura Y, Hamamoto T, Watanabe A, Yaguchi T, Sano T, Suematsu R, Kato Y, Miyata J, et al. Mixed Infection of Cytomegalovirus and Pulmonary Nocardiosis Caused by Nocardia elegans Diagnosed Using Nanopore Sequencing Technology. Intern Med (2022) 61:1613–1617. doi: 10.2169/internalmedicine.7639-21

63. Wu MY-C, Tsai TC, Hsieh C-H, Barbosa F, Somers A. Unmasking Pulmonary Nocardiosis in an Asthmatic Host Presenting With Chronic Cough, Pulmonary Nodularity, and Ground-Glass Opacities. Cureus (2025) doi: 10.7759/cureus.84739

64. Wu X-K, Lin Q. Pulmonary alveolar proteinosis complicated with nocardiosis: A case report and review of the literature. WJCC (2021) 9:2874–2883. doi: 10.12998/wjcc.v9.i12.2874

65. Xiao Y, Wang H, Tian T, Zheng J, Liu M, Wang Q, Li J. Liver cirrhosis complicated with pulmonary Nocardia infection: A case report and literature review. Medicine (2024) 103:e40054. doi: 10.1097/MD.0000000000040054

66. Yagyu K, Nakatsuji Y, Matsushita H. Elevated serum β -d- glucan levels in cavitary pulmonary nocardiosis. BMJ Case Rep (2020) 13:e234738. doi: 10.1136/bcr-2020-234738

67. Yan C, Liu T-T, Gao L-T. Chronic inflammatory demyelinating polyneuropathy with pulmonary nocardiosis: A case report. Medicine (2024) 103:e38544. doi: 10.1097/MD.0000000000038544

68. Zuo H, Ye J, Li C, Li S, Gu J, Dong N, Zhao Y, Hao J, Song M, Guo Y, et al. Myasthenia gravis complicated with pulmonary infection by Nocardia cyriacigeorgica: a case report and literature review. Front Med (2024) 11:1423895. doi: 10.3389/fmed.2024.1423895

69. 蔺晨, 王世寿, 安然, 冯涛, 黄诗玫. 肺部豚鼠耳炎诺卡菌合并曲霉感染1例. Zhonghua Jie He He Hu Xi Za Zhi (2024) 47:237–240. doi: 10.3760/cma.j.cn112147-20230714-00008

70. Abdel-Rahman N, Izhakain S, Wasser WG, Fruchter O, Kramer MR. Endobronchial enigma: A clinically rare presentation of nocardia beijingensis in an immunocompetent patient. Case Rep Pulmonol (2015) 2015:970548. doi: 10.1155/2015/970548

71. Aggarwal D, Garg K, Chander J, Saini V, Janmeja AK. Pulmonary nocardiosis revisited: A case series. Lung India (2015) 32:165–168. doi: 10.4103/0970-2113.152638

72. Canterino J, Paniz-Mondolfi A, Brown-Elliott BA, Vientos W, Vasireddy R, Wallace RJ, Campbell S. Nocardia thailandica pulmonary nocardiosis in a post-solid organ transplant patient. J Clin Microbiol (2015) 53:3686–3690. doi: 10.1128/JCM.00959-15

73. Castellana G, Grimaldi A, Castellana M, Farina C, Castellana G. Pulmonary nocardiosis in chronic obstructive pulmonary disease: a new clinical challenge. Respir Med Case Rep (2016) 18:14–21. doi: 10.1016/j.rmcr.2016.03.004

74. Ercibengoa Arana M, Marimón Ortiz de Zarate JM. First report of nocardia fusca isolated in humans. BMJ Case Rep (2015) 2015:bcr2015209538. doi: 10.1136/bcr-2015-209538

75. Garcia RR, Bhanot N, Min Z. A mimic’s imitator: a cavitary pneumonia in a myasthenic patient with history of tuberculosis. BMJ Case Rep (2015) 2015:bcr2015210264. doi: 10.1136/bcr-2015-210264

76. Hakim H, Rao NN, Faull RJ, Coates PT. Nocardiosis presenting as a lung mass in a kidney transplant recipient. Nephrology (2015) 20:6–9. doi: 10.1111/nep.12420

77. Han T, Liu Y-M, Yang T, Dai H-P, Zhang X-L. A novel case of pulmonary nocardiosis with secondary hemophagocytic lymphohistiocytosis. Chin Med J (Engl) (2017) 130:2128–2129. doi: 10.4103/0366-6999.213421

78. Jayaschandran V, Gjorgova-Gjeorgjievski S, Siddique H. Pulmonary nocardiosis in a patient with idiopathic CD4 T-lymphocytopenia. Respirol Case Rep (2017) 6:e00283. doi: 10.1002/rcr2.283

79. Kato K, Noguchi S, Naito K, Ikushima I, Hanaka T, Yamasaki K, Kawanami T, Yatera K. Pulmonary nocardiosis caused by nocardia exalbida in a patient with lung cancer and radiation pneumonitis: a case report and literature review. Intern Med (2019) 58:1605–1611. doi: 10.2169/internalmedicine.2177-18

80. Khadka P, Basnet RB, Rijal BP, Sherchand JB. Pulmonary nocardiosis masquerading renascence of tuberculosis in an immunocompetent host: a case report from Nepal. BMC Res Notes (2018) 11:488. doi: 10.1186/s13104-018-3604-2

81. Kibe S, Meigh R, Moon T, Kastelik J, Morjaria J. Nocardia cyriacigeorgica in an immunocompetent patient. Ther Adv Respir Dis (2015) 9:28–30. doi: 10.1177/1753465814565352

82. Kuchibiro T, Ikeda T, Nakanishi H, Morishita Y, Houdai K, Ito J, Gonoi T. First case report of pulmonary nocardiosis caused by nocardia mexicana. JMM Case Rep (2016) 3:e005054. doi: 10.1099/jmmcr.0.005054

83. Kumar A, Reddy A, Satagopan K. Unusual presentation of pulmonary nocardiosis as pyopneumothorax in HIV. Lung India (2015) 32:295–296. doi: 10.4103/0970-2113.156261

84. Liu C, Feng M, Zhu J, Tao Y, Kang M, Chen L. Severe pneumonia due to nocardia otitidiscaviarum identified by mass spectroscopy in a cotton farmer. Medicine (Baltimore) (2017) 96:e6526. doi: 10.1097/MD.0000000000006526

85. Manoharan H, Selvarajan S, Sridharan KS, Sekar U. Pulmonary infections caused by emerging pathogenic species of nocardia. Case Rep Infect Dis (2019) 2019:5184386. doi: 10.1155/2019/5184386

86. Martinez-Gamboa A, Cervera-Hernandez ME, Torres-Gonzalez P, Rangel-Cordero A, Ponce-de-Leon A, Sifuentes-Osornio J. First case of nocardia amamiensis pulmonary infection in Mexico. New Microbes New Infect (2016) 16:1–2. doi: 10.1016/j.nmni.2016.12.005

87. Matsubayashi S, Iikura M, Numata T, Izumi S, Sugiyama H. A case of aspergillus and nocardia infections after bronchial thermoplasty. Respirol Case Rep (2018) 7:e00392. doi: 10.1002/rcr2.392

88. Mendonca NP, Kadayakkara DK, Forde IC, Rudkovaskaia A, Saul ZK, Lobo DJ. Pulmonary nocardiosis in a multiple myeloma patient treated with proteasome inhibitors. Am J Case Rep (2016) 17:76–78. doi: 10.12659/AJCR.896280

89. Miyaoka C, Nakamoto K, Shirai T, Miyamoto M, Sasaki Y, Ohta K. Pulmonary nocardiosis caused by nocardia exalbida mimicking lung cancer. Respirol Case Rep (2019) 7:e00458. doi: 10.1002/rcr2.458

90. Mylonas CC, Gomatou G, Asimakopoulou A, Masaoutis C, Kyriakopoulos G, Kopelia M, Syrigos K, Poulakou G. Pulmonary nocardiosis associated with cushing’s disease: a case report. Monaldi Arch Chest Dis (2019) 89:1130. doi: 10.4081/monaldi.2019.1130

91. Narula N, Bourne M, Bhagra A. Immunosuppression and a serious opportunistic infection: an unfortunate price to pay. BMJ Case Rep (2015) 2015:bcr2014207712. doi: 10.1136/bcr-2014-207712

92. Okawa S, Sonobe K, Nakamura Y, Nei T, Kamio K, Gemma A. Pulmonary nocardiosis due to nocardia asiatica in an immunocompetent host. J Nippon Med Sch (2015) 82:159–162. doi: 10.1272/jnms.82.159

93. Onaiwu CO, Velagapudi M, Sarsam L, Utley L, Bricker L, Bendi VS, Vivekanandan R. Rare multidrug-resistant pulmonary nocardiosis in AIDS. Cureus 9:e1839. doi: 10.7759/cureus.1839

94. Pannu S, Pannu AK. Primary pulmonary nocardiosis by nocardia brasiliensis: a case report and review of indian literature. J Fam Med Prim Care (2019) 8:3035–3038. doi: 10.4103/jfmpc.jfmpc_576_19

95. Sadamatsu H, Takahashi K, Tashiro H, Komiya K, Nakamura T, Sueoka-Aragane N. Successful treatment of pulmonary nocardiosis with fluoroquinolone in bronchial asthma and bronchiectasis. Respirol Case Rep (2017) 5:e00229. doi: 10.1002/rcr2.229

96. Schoen L, Santoro JD, Milla C, Bhargava S. Pulmonary nocardiosis in an immunocompetent patient with cystic fibrosis. Case Rep Pulmonol (2015) 2015:984171. doi: 10.1155/2015/984171

97. Shahani L. Utility of transbronchial lung biopsy in diagnosis of cavitary lung lesion in a patient with HIV infection. BMJ Case Rep (2015) 2015:bcr2015209726. doi: 10.1136/bcr-2015-209726

98. Sheikh-Taha M, Corman LC. Pulmonary nocardia beijingensis infection associated with the use of alemtuzumab in a patient with multiple sclerosis. Mult Scler J (2017) 23:872–874. doi: 10.1177/1352458517694431

99. Shokri D, Motalebirad T, Jafarinia M, Azadi D, Ghaffari K. First case report of pulmonary and cutaneous nocardiosis caused by nocardia mexicana in Iran. Access Microbiol (2019) 1:e000016. doi: 10.1099/acmi.0.000016

100. Singh I, West FM, Sanders A, Hartman B, Zappetti D. Pulmonary nocardiosis in the immunocompetent host: case series. Case Rep Pulmonol (2015) 2015:314831. doi: 10.1155/2015/314831

101. Singh AK, Shukla A, Bajwa R, Agrawal R, Srivastwa N. Pulmonary nocardiosis: unusual presentation in intensive care unit. Indian J Crit Care Med : Peer-rev Off Publ Indian Soc Crit Care Med (2018) 22:125–127. doi: 10.4103/ijccm.IJCCM_472_17

102. Soares D, Reis-Melo A, Ferraz C, Guedes Vaz L. Nocardia lung abscess in an immunocompetent adolescent. BMJ Case Rep (2019) 12:bcr-2018-227499. doi: 10.1136/bcr-2018-227499

103. Tamakoshi J, Kimura R, Takahashi K, Saito H. Pulmonary reinfection by nocardia in an immunocompetent patient with bronchiectasis. Intern Med (2018) 57:2581–2584. doi: 10.2169/internalmedicine.0531-17

104. Thirouvengadame S, Muthusamy S, Balaji VK, Easow JM. Unfolding of a clinically suspected case of pulmonary tuberculosis. J Clin Diagn Res : JCDR (2017) 11:DD01–DD03. doi: 10.7860/JCDR/2017/25788.10404

105. Trastoy R, Manso T, García X, Barbeito G, Navarro D, Rascado P, Pérez Del Molino ML. [pulmonary co-infection due to nocardia cyriacigeorgica and aspergillus fumigatus]. Rev Esp Quimioter: Publ Of Soc Esp Quimioter (2017) 30:123–126.

106. Verma R, Walia R, Sondike SB, Khan R. Pulmonary nocardiosis in an adolescent patient with crohn’s disease treated with infliximab: a serious complication of TNF-alpha blockers. W V Med J (2015) 111:36–39.

107. Weber M, Rüddel J, Bruns T, Pletz M, Stallmach A. Pulmonary co-infection with nocardia species and nontuberculous mycobacteria mimicking miliary tuberculosis in a patient with crohn’s disease under combined immunosuppressive therapy. Z Gastroenterol (2018) 56:569–572. doi: 10.1055/a-0614-2871

108. Wu J, Wu Y, Zhu Z. Pulmonary infection caused by nocardia cyriacigeorgica in a patient with allergic bronchopulmonary aspergillosis. Medicine (Baltimore) (2018) 97:e13023. doi: 10.1097/MD.0000000000013023

109. Yu S, Wang J, Fang Q, Zhang J, Yan F. Specific clinical manifestations of nocardia: a case report and literature review. Exp Ther Med (2016) 12:2021–2026. doi: 10.3892/etm.2016.3571
